# Understanding Optical Properties and Electronic Structures of High‐Entropy Alloyed Perovskite Nanocrystals

**DOI:** 10.1002/anie.202505890

**Published:** 2025-07-24

**Authors:** Yung‐Tai Chiang, Sunil B. Shivarudraiah, Alexander Wieczorek, Khoong Hong Khoo, Zhidong Leong, Jia Wei Melvin Lim, Zengshan Xing, Sudhir Kumar, Simon F. Solari, Yen‐Ting Li, Yu‐Cheng Chiu, Tze Chien Sum, Yun Liu, Sebastian Siol, Chih‐Jen Shih

**Affiliations:** ^1^ Institute for Chemical and Bioengineering, ETH Zürich Zürich 8093 Switzerland; ^2^ Laboratory for Surface Science and Coating Technologies Empa – Swiss Federal Laboratories for Materials Science and Technology Dübendorf 8600 Switzerland; ^3^ Institute of High Performance Computing (IHPC) Agency for Science, Technology and Research (A*STAR) 1 Fusionopolis Way, 16‐16 Connexis Singapore 138632 Republic of Singapore; ^4^ Division of Physics and Applied Physics, School of Physical and Mathematical Sciences Nanyang Technological University 21 Nanyang Link Singapore 637371 Singapore; ^5^ Department of Chemical Engineering National Taiwan University of Science and Technology Taipei 10607 Taiwan; ^6^ National Synchrotron Radiation Research Center Hsinchu 30076 Taiwan; ^7^ Advanced Research Center for Green Materials Science and Technology National Taiwan University Taipei 10617 Taiwan

**Keywords:** High‐entropy perovskites, Radiative lifetime, Shallow states, Symmetry breaking

## Abstract

High‐entropy alloying (HEA) has emerged as a prominent strategy to modulate physiochemical properties of nanomaterials. Nevertheless, this approach is underexplored in luminescent semiconductor nanocrystals (NCs) due to the lack of understanding into the HEA‐induced electronic effect and photophysical behaviors. Herein, harnessing the defect tolerance of metal halide perovskite NCs, we systematically synthesized and characterized high‐entropy halide perovskite (HEP) NCs containing multiple B‐site elements (Pb^2+^, Sr^2^⁺, Ca^2^⁺, Cd^2^⁺, and Mg^2^⁺). High‐resolution transmission electron microscopy, transient photoluminescence and absorption spectroscopy, X‐ray photoemission spectroscopy, and density functional theory simulations are employed to unravel the evolution of electronic structures with respect to the alloying degree and link them to the spectral signatures and photostability. Counterintuitively, although the HEP NCs exhibit lateral sizes smaller than the Bohr diameter of CsPbBr_3_ NCs (∼7 nm), HEA reduces the band dispersion and broadens the conduction band, thereby vanishing the excitonic feature by forming near band‐edge shallow states. We show that these HEA‐induced shallow states foster rapid radiative recombination and improve photostability, accompanied by a significantly reduced lead content by up to 70%. These findings pioneer the understanding of the correlation between HEA‐induced electronic effect and photophysical properties, highlighting the versatility of HEA for band structure engineering and stabilization of metal halide perovskites NCs.

## Graphical abstract

High entropy perovskite (HEP) nanocrystals (NCs) are synthesized and their anomalous optical and electronic signatures are characterized by temperature‐dependent photophysical measurements, electron microscopy, X‐ray photoelectron spectroscopy, and density functional theory. Despite the smaller size of HEP NCs than the Bohr diameter of pristine NCs, they possess wider bandgaps and featureless band structures due to the formation of shallow states. In situ luminescence analysis further demonstrates the superior photostability of HEP NCs thin films.

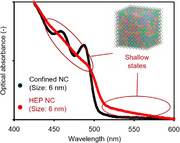

## Introduction

The discovery of multi‐component alloys has generated considerable research interests across various material systems, including ceramics, semiconductor oxides, and metals owing to the extensive compositional space and unique functionalities derived from the large configurational entropy.^[^ ^1^, ^2^, ^3^, ^4^ ^]^ The incorporation of more than four primary and secondary elements into a single‐phase solid solution is able to unlock novel physiochemical properties, such as exceptional thermal stability, improved wear resistance, and unique electronic features, rendering them attractive for aerospace, automotive, energy, and catalysis applications.^[^ ^5^, ^6^, ^7^, ^8^, ^9^ ^]^ Analogous to bulk high‐entropy alloys, which typically require high‐temperature synthesis, high‐entropy nanoparticles have also showcased promising results by exploiting colloidal ligand chemistry and different coordinating solvents.^[^ ^10^, ^11^, ^12^ ^]^ Although phase segregation is generally thermodynamically favorable, efficient strategies such as configuration of elements, precise composition control, and judicious selection of surfactants, are capable of stabilizing high‐entropy nanoparticles at metastable states.^[^ ^13^, ^14^, ^15^ ^]^ Accordingly, several high‐entropy nanoparticle systems have been reported, for instance intermetallics,^[^ ^16^, ^17^ ^]^ metal sulfides,^[^ ^18^, ^19^ ^]^ metal oxides,^[^ ^20^ ^]^ and metal oxysulfides.^[^ ^21^ ^]^ Nevertheless, there are limited reports deploying the high‐entropy concept in fluorescent colloidal nanocrystals (NCs) for optoelectronic applications, likely because the extensive alloying process introduces crystalline defects and suppresses efficient excitonic recombination, thereby lowering the quantum efficiency.^[^ ^22^, ^23^, ^24^ ^]^

In this regard, the advancement of lead halide perovskite (LHP) NCs enlightens a new framework for HEA owing to the intrinsic defect tolerance nature and high photoluminescence quantum yields (*η*_PL_).^[^ ^25^, ^26^, ^27^, ^28^ ^]^ Theoretically, the incorporation of multiple elements could be achieved by substituting the B‐site atom with multiple divalent ions in the perovskite ABX_3_ crystal lattice. Indeed, although the partial replacement of B‐site Pb^2+^ ions by one or two metal cations has been extensively investigated, with primary efforts dedicated to uncover the roles of surface defects^[^ ^29^, ^30^, ^31^, ^32^ ^]^ and trap states,^[^ ^33^, ^34^, ^35^, ^36^ ^]^ fundamental understanding of B‐site HEA effect and their link to photophysical properties still remains elusive. Since the electronic structure is dictated by the B‐site valence shells, it is expected that the change in orbital hybridization will modulate the effective mass of excitons and induce lattice symmetry breaking.^[^ ^37^, ^38^ ^]^ Previously, we reported a solid‐phase synthesis protocol for the preparation of HEP NCs based on methylammonium lead bromide (MAPbBr_3_) NCs, leading to a substantial reduction of lead content by up to 55% and near‐unity *η*_PL_.^[^ ^39^ ^]^ Similarly, HEA‐based perovskite NCs were synthesized via a rapid room‐temperature process,^[^ ^40^ ^]^ and optimized to achieve highly efficient and spectrally stable electroluminescence.^[^ ^41^ ^]^ Despite significant progresses, it remains unclear that how HEA rectifies the electronic structure of LHP NCs and modulate their excitonic behaviors, which is crucial to unlock their full potential and realize advanced optoelectronic applications. Herein, combining high‐resolution transmission electron microscopy, transient photoluminescence and absorption spectroscopy, X‐ray photoemission spectroscopy, and density functional theory (DFT) simulations, we narrow the gap between the understanding of electronic structures and novel optical properties of HEP NCs.

## Results and Discussion

### Structural and Optical Characteristics of HEP NCS

**Figure 1:**
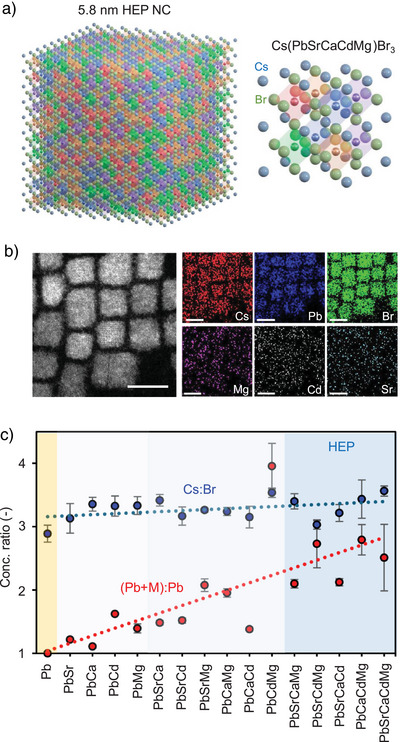
Concept of HEP NCs. a) Schematic illustration of a B‐site HEP NC containing approximately 1000 [MBr_6_]^4–^ octahedral unit cells. b) Representative HRSTEM EDS map for Cs(PbSrCdMg)Br_3_ HEP NCs, revealing that the alloying elements, Mg, Cd, and Sr, are randomly distributed within individual NCs. Scale bars: 5 nm. c) Statistical analysis for the EDS‐analyzed Cs:Br and (Pb + M):Pb ratios for all NC samples considered in this study. The toxic Pb^2+^ could be largely reduced by up to 70% using our HEP protocol.

B‐site HEP NCs were systematically prepared through ligand‐assisted multi‐cationic exchange of CsPbBr_3_ NCs. In brief, CsPbBr_3_ NCs were first synthesized using a ligand‐assisted reprecipitation protocol.^[^ ^42^ ^]^ Subsequently, the colloidal solutions were mixed with excess divalent metal bromide salts (MBr_2_, where M^2+^ = Sr^2+^, Ca^2+^, Cd^2+^, Mg^2+^) partially dissolved in nonpolar toluene via the addition of amphiphilic ligands (details see Supplementary Section 1.2). Due to the soft ionic lattice nature of lead halide perovskites, the partial and gradual substitution of Pb^2+^ with ligand‐stabilized M^2+^ gradually became entropically favorable,^[^ ^32^ ^]^ thus forming the B‐site HEP NCs. Figure 1a presents a schematic diagram illustrating a HEP NC containing approximately 1000 [MBr_6_]^4–^ octahedral unit cells, in which M^2+^ ions are embedded in the lattice through HEA. For each colloidal NC sample synthesized, we prepared solid thin films by drop‐casting the solution onto silicon substrates, followed by extensive energy‐dispersive X‐ray spectroscopy (EDS) in a scanning electron microscope (SEM) or on TEM grids using high‐resolution scanning transmission electron microscopy (HR‐STEM) (Figures S1–S5 and Table S1). Figure 1b shows a representative HR‐STEM EDS mapping result for Cs(PbSrCdMg)Br_3_ HEP NCs, highlighting the alloying elements, Mg, Cd, and Sr, are randomly distributed within individual NCs, without phase separation or metal segregation.

Our systematic SEM EDS measurements enable quantitative estimation of atomic ratios Cs:Br and (Pb + M):M for all synthesized samples (Figures 1c, S6–S8, and Table S2). The Cs:Br ratio nicely aligns with the expected perovskite stoichiometry of 3, showing that our synthetic protocol did not alter the A‐site composition significantly and removed excessive metal bromides from the crude solution (Table S3). Alternatively, despite a degree of variation, there exists a clear trend that the (Pb + M):M ratio increases substantially with more secondary elements, which is driven by the increasing configurational entropy of the systems. For simplification, the B‐site elements are used to denote each HEP NC synthesized in this work. We discovered that the majority of secondary M^2+^ ions, especially the smaller Cd^2+^ and Mg^2+^ ions, replaced the B‐site Pb^2+^ ions, which aligns with the substitutional mechanism proposed by Phung et al. in monodoped perovskite NCs.^[^ ^43^ ^]^ Importantly, our results indicate that the toxic Pb^2+^ could be effectively reduced by up to 70% using our protocol, rendering the commercial applications of the HEP NCs much more attractive.

**Figure 2:**
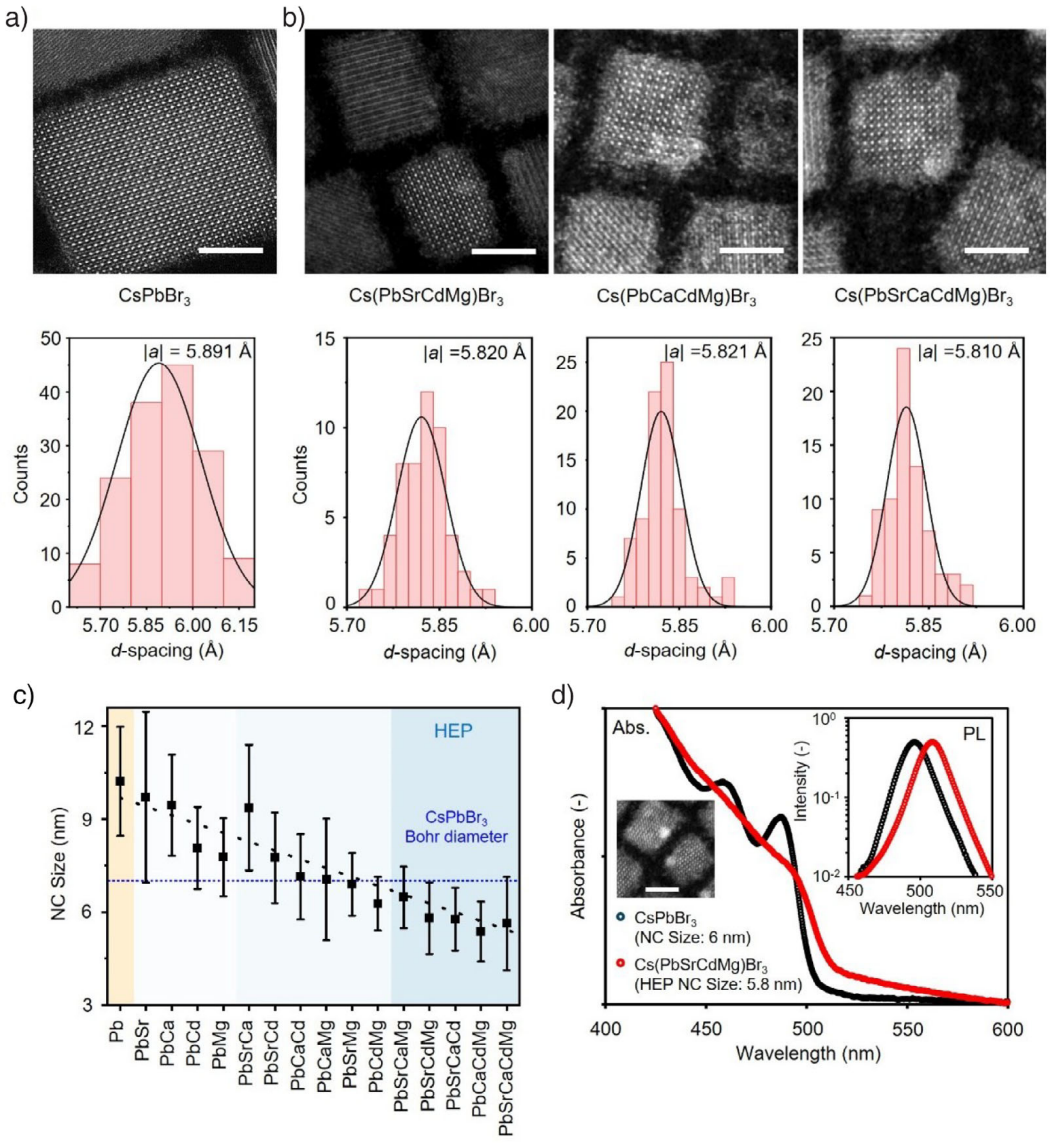
Structural and crystallographic properties of HEP NCs. a,b) HRSTEM images (top) and extracted lattice parameters (bottom) for a) the parent CsPbBr_3_ and b) HEP NCs. Upon HEA, the perovskite lattice contracts resulting from doping of small‐radii B‐site M^2+^ ions. Scale bars: 5 nm. c) Statistical analysis for the NC lateral sizes extracted from the TEM images of synthesized NCs, showing a reduction of size. In particular, HEP NCs of B‐site alloying of more than three extra ions decreases the size below the Bohr diameter of CsPbBr_3_ (∼ 7 nm). d) Comparison of absorption (Abs.) and photoluminescence (PL; right inset) spectra for HEP (average NC size: 5.7 nm; red curves) and quantum‐confined CsPbBr_3_ (average NC size: ∼ 6 nm; black curves) NCs, showing a lack of excitonic feature for HEP NCs. Left inset: HRSTEM image for the quantum‐confined CsPbBr_3_ NCs. Scale bar: 5 nm.

We examined the structural and crystallographic properties of HEP NCs (Figure 2). HR‐STEM is used to analyze and quantify the crystallographic profiles of parent CsPbBr_3_ and three representative HEP NCs, including Cs(PbSrCdMg)Br_3_, Cs(PbCaCdMg)Br_3_, and Cs(PbSrCaCdMg)Br_3_ (Figure 2a, b). The atomically resolved images allow us to precisely quantify the lattice parameter *d*‐spacing, |*a*|, exhibiting a reduction from 5.89 to 5.81 Å upon HEA. These findings agree well with the smaller ionic radii of the doping cations compared to Pb^2+^. We did not observe phase separation within individual HEP NCs, echoing the previous EDS results. The corresponding X‐ray diffraction (XRD) crystallographs suggest that all the synthesized HEP NCs inherit the parent orthorhombic structure (Figure S9), with a slight degree of lattice contraction consistent with the HRSTEM observation (Figure S10). Interestingly, upon HEA, in addition to lattice contraction, statistical TEM analysis revealed that the lateral size of the synthesized NCs substantially decreased from 10 nm to approximately 5.5 nm for the quaternary and quinary HEP NCs, while possessing high monodispersity (Figures 2c and S11). We attribute the observed NC size reduction to i) the addition of excess ligands and metal bromides in the synthetic protocol, and ii) re‐assembly of M‐site octahedral unit cells.^[^ ^32^, ^44^, ^45^, ^46^, ^47^ ^]^ We would like to point out that the average size for the quaternary and quinary HEP NCs are all below 6 nm, considerably smaller than the Bohr diameter (*d*_B_) of CsPbBr_3_ (∼7 nm).^[^ ^48^ ^]^

Optical characterizations are then employed to investigate the steady state absorption and emission spectra for the colloidal HEP NCs (Figures S12–S14). Surprisingly, although the small NC sizes should give rise to a strong quantum‐confined nature, all absorption spectra lack excitonic feature and showcase a smooth bandgap edge. For comparison, we synthesized the quantum‐confined CsPbBr_3_ NCs with an average NC size of ∼6 nm (HRSTEM image, see Figure 2d inset). Figure 2d compares the absorption spectra between the 5.7 nm Cs(PbSrCdMg)Br_3_ HEP and the 6 nm CsPbBr_3_ NCs. Clearly, in addition to the lack of discernable features of confined resonance, the Cs(PbSrCdMg)Br_3_ HEP NCs exhibit a smaller hypsochromic shift (the emission wavelength λ_PL_ = 506 nm). Furthermore, the long‐tail absorption at the band edge, together with the slightly stoke‐shifted photoemission, suggest the presence of shallow states upon HEA.^[^ ^49^, ^50^, ^51^ ^]^ We noticed that these optical behaviors are in stark contrast with the quantum‐confined CsPbBr_3_ NCs, where a larger bandgap alongside a strong absorption peak locating at 475 nm are present due to the quantized density of states (DOS).^[^ ^52^ ^]^ Note that we had confirmed the extended absorption tail does not result from the optical scattering effects, as we filtered the colloidal solution through a porous 0.2 µm polytetrafluoroethylene (PTFE) filter and excited the solution at the tail wavelength region (Figure S15). Notably, when PbBr_2_ alone was introduced during the cationic exchange, quantum‐confined nanoplatelets were formed via structural deterioration, underscoring the distinct role of B‐site HEA in modulating photophysical properties (Figure S16). Our findings clearly indicate that the electronic configuration of the small HEP NCs behave closer to bulk semiconductors rather than confined quantum dots (QDs); in other words, the classical quantum confinement fails to describe the unique photoelectronic properties of HEP NCs. Detailed characterizations are then performed to unravel the mechanisms governing the anomalous optical behaviors.

### Shallow States‐Assisted Rapid Photoemission

**Figure 3:**
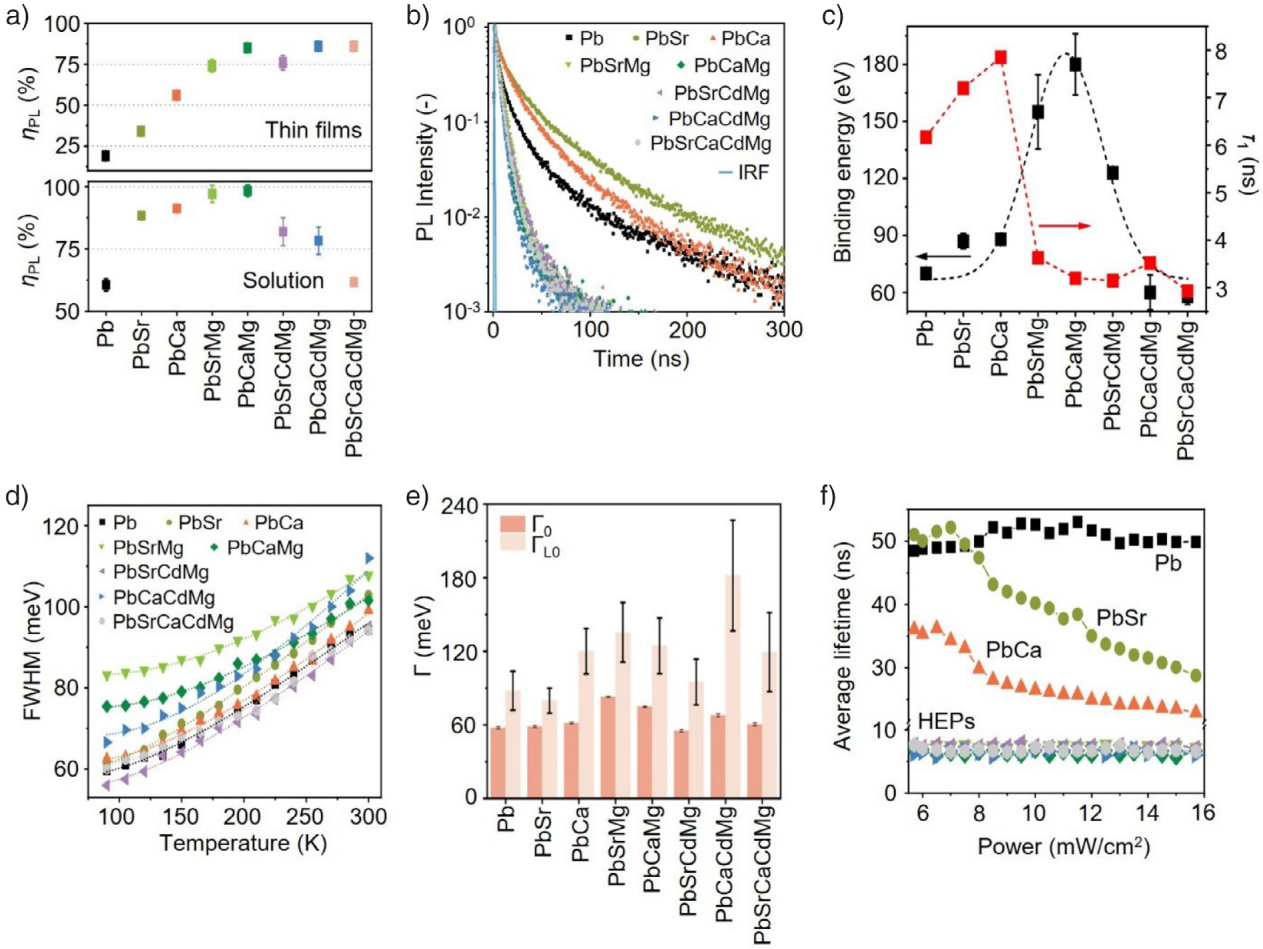
Photophysical characterization of HEP NCs. Room‐temperature a) solution and thin‐film *η*_PL_ and b) TRPL responses for HEP NCs. c) The exciton binding energy extracted from variable temperature PL measurements and the prompt radiative lifetime component *τ*_1_ characterized at room temperature, with standard deviation indicated in the figure. d) The variation of emission FWHM values characterized at low temperatures. e) The extracted peak broadening contributions from lattice imperfection (Г_0_) and longitudinal phonon scattering (Г_LO_) by fitting with the Fröhlich equation, with standard deviation provided in the figure. f) Extracted exciton lifetimes at room temperature with respect to excitation power for the HEP NCs.

Figure 3 presents the photophysical properties of HEP NCs. Compared to the parent CsPbBr_3_ NCs, the solution and thin‐film *η*_PL_ are substantially enhanced in alloyed NCs, attaining the highest values for Cs(PbCaMg)Br_3_ NCs (∼90% and ∼100% for thin films and solutions, respectively; see Figure 3a). We attribute the enhanced solution *η*_PL_ to the defects passivation from the excess bromides and amphiphilic ligands on the NC surfaces, as reflected by the increased Br/Cs ratios in HEP NCs (Figure 1c) and the slight *η*_PL_ enhancement upon the addition of excess ligands (Figure S17). Nevertheless, for HEP NCs, the solution PL quenches slightly in the quaternary and quinary B‐site systems, presumably owing to trap states introduced by extensive alloying.^[^ ^53^ ^]^ Interestingly, the HEP NC thin‐film samples universally attain high *η*_PL_ of >90%, which is attributed to the ligand adsorption/desorption equilibrium and NC self‐assembly minimizing the non‐radiative pathways^[^ ^54^ ^]^ and the aggregation‐induced emission due to restricted motion of surface ligands.^[^ ^55^ ^]^ This is confirmed qualitatively by close inspection of the SEM images and quantitatively by grazing incident small‐angle XRD of HEP NC films, showcasing the formation of self‐assembled superlattices that fosters long‐range packing and reduces the number of undercoordinated bonds (Figures S18, S19).^[^ ^56^ ^]^ Hereafter, the photophysical characterization is based on the thin‐film samples. The boosted radiative recombination efficiency in HEP NCs reaffirms a distinct emission mechanism from quantum‐confined NCs, where a significantly low *η*_PL_ are observed (*η*_PL _= 30%).^[^ ^48^, ^57^ ^]^

To unravel the carrier recombination dynamics in HEP NCs, we performed room‐temperature time‐resolved (TR) PL spectroscopy of HEP NCs thin films (Figure 3b). Interestingly, according to the enhanced *η*_PL_ relative to parent CsPbBr_3_ NCs, one might anticipate elongated lifetimes induced by defect or surface passivation.^[^ ^58^, ^59^, ^60^, ^61^ ^]^ However, in all HEP NC species considered here, the average PL lifetime significantly decreased by up to nearly one order of magnitude, from 48.4 ns to as low as 6.1 ns upon HEA (Table S4). A similar PL lifetime reduction is also observed in the quantum‐confined CsPbBr_3_ NCs, suggesting that size effect cannot be ignored (Figure S20 and Table S4). To explore different radiative channels and their contributions, we examined the TRPL decay at cryogenic temperatures between 77 and 300 K, in which the trap states are mitigated to allow the intrinsic recombination process to dominate.^[^ ^62^, ^63^ ^]^ Each TRPL response was fitted with a bi‐ or tri‐exponential function, where the most rapid component conforms to the band‐edge radiative recombination at low temperatures, while the second and third components can be assigned to shallow and deep trap‐assisted recombination, respectively.^[^ ^64^, ^65^ ^]^ Although the enhanced exciton binding energy at lower temperature will prompt rapid radiative recombination in luminescent NCs, we observed that, surprisingly, the average PL lifetimes of HEP NCs are reduced by a factor of 18 when the temperature decreases from 300 to 77 K (Figure S20 and Table S5), whereas that for parent CsPbBr_3_ and single‐doped NCs only decreased by two folds. Because the defects‐/deep‐states‐mediated pathways are quenched at low temperatures, the drastic lifetime reduction in HEP NCs indicates that the band edge coupled to shallow states‐mediated radiative channels are substantially accelerated than parent and single‐alloyed species. We can therefore infer that the shallow states introduced in HEA are responsible for the rapid electron‐hole recombination observed here.^[^ ^66^, ^67^ ^]^

To further strengthen our hypothesis, temperature‐dependent PL intensities are analyzed (Figures S22–S24), allowing us to extract the exciton binding energy using the Arrhenius equation.^[^ ^68^, ^69^, ^70^ ^]^ We found that trinary perovskite NCs, such as Cs(PbCaMg)Br_3_, yield the highest binding energy up to 180 meV, as compared to 70 meV in parent CsPbBr_3_ NCs (Figure 3c). Remarkably, this exceptionally high binding energy is typically found only in strongly confined nanocrystals.^[^ ^71^ ^]^ As the trinary NC sizes remain larger than the Bohr diameter parent CsPbBr_3_ NCs (see Figure 2c), and the absorption spectra lack the excitonic feature (see Figure S12), we attribute the enhanced band‐edge recombination to the high binding energy, endorsing the extremely short prompt radiative lifetime value at room temperature (∼3 ns). However, excessive alloying in HEP NC systems reverses the trend, decreasing the binding energy, despite the confinement effect resulting from the NC size reduction (Figure 2c). We hypothesize that excessive alloying induces wavefunction delocalization that disrupts the recombination process. This highlights the concomitant existence of strong exciton localization induced by size reduction and HEA in HEP NCs.^[^ ^72^, ^73^ ^]^

Indeed, our synthesized HEP NCs are not “defect‐free”. We characterized the PL full width half maximum (FWHM) at various temperatures and fitted with the Fröhlich equation (Figure 3d, e).^[^ ^74^ ^]^ The relatively higher values for the extracted peak broadening contribution from lattice imperfection, Г_0_, suggest the increase of defective states upon HEA. To further elucidate the key role of defects, Figure 3f compares the extracted average radiative lifetimes with respect to the pumping laser power. Among the NC samples considered here, two distinct trends were identified: lifetimes of parent CsPbBr_3_ NCs remained approximately constant and independent of the laser power, while those for binary alloyed NCs decrease with increasing laser power. The lifetime decrease at high laser powers, according to previous reports,^[^ ^75^ ^]^ results from the populated trapped states that promote the bimolecular exciton‐exciton annihilation at high laser powers. In HEP NCs, however, this effect appears to be counter‐balanced, exhibiting power‐independent lifetimes (Figure 3f). Echoing the observed binding energy decrease in HEP NCs (Figure 3c), the exciton localization and suppressed diffusion pathways may suppress the many‐body recombination processes upon HEA.^[^ ^76^, ^77^ ^]^

**Figure 4:**
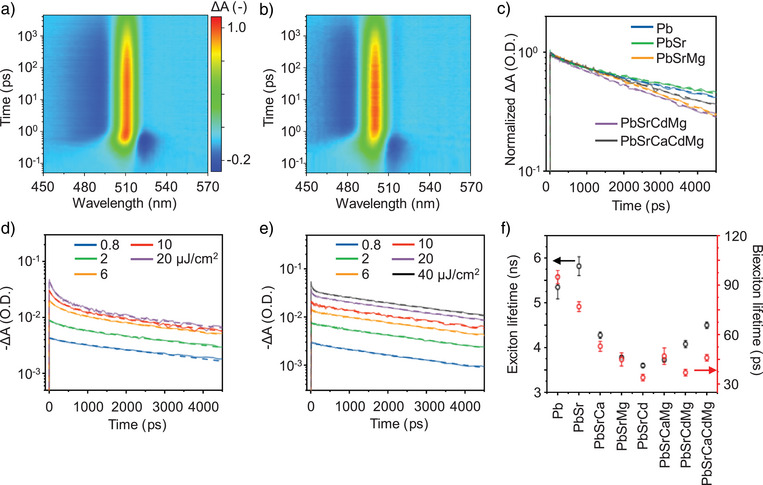
Transient absorption (TA) responses of HEP NCs. Representative normalized TA spectra for a) parent CsPbBr_3_ and b) Cs(PbSrCdMg)Br_3_ NCs excited at 400 nm. c) Comparison of GSB kinetics for NC samples with different doping elements at low pump fluence of ∼0.8 µJ cm^−2^. Pump power‐dependent GSB kinetics for d) CsPbBr_3_ and e) Cs(PbSrCdMg)Br_3_ NCs. The dashed curves correspond to fittings of tri‐exponential functions convoluted with instrument response function. f) Extracted exciton and biexciton lifetimes from the pump power‐dependent GSB kinetics.

We further investigated the non‐radiative charge carrier dynamics of HEP NCs using the transient absorption (TA) spectroscopy. Figure 4a, b show the representative TA spectra for the parent CsPbBr_3_ and Cs(PbSrCdMg)Br_3_ HEP NCs in solutions, exhibiting a dominant positive signal corresponding to the ground state bleaching (GSB) at the band edge wavelengths of 516 and 507 nm, respectively. Figure 4c compares the GSB kinetics that characterize the excited‐state carrier dynamics for a number of NC samples considered here. Overall, the samples with higher numbers of alloying elements exhibit shorter carrier lifetimes. The GSB kinetics were further probed at various pumping powers (Figures 4d, e, and S25), followed by globally fitting with the triexponential functions with the instrument response function deconvolved (Figure 4f and Table S6). The kinetics at low fluence demonstrates a mono‐exponential decay, characterizing the exciton lifetime τ_1_. In contrast, the τ_2_ and τ_3_ appear at higher pump fluences, attributed to the multiexciton processes of biexcitons and trions, respectively.^[^ ^78^ ^]^ The obtained lifetimes of biexcitons (tens of picosecond) and trions (hundreds of picosecond) are in line with their reported time scales in halide perovskite NCs. ^[^ ^78^, ^79^, ^80^ ^]^

Overall, the exciton and biexciton lifetimes become shorter upon metal alloying. Although one could attribute the observation to the NC size reduction in HEP NCs (Figures 2c and S26), we noticed distinct trends for exciton and biexciton lifetimes upon alloying with multiple metal elements, highlighting the role of alloying apart from the size effect. Specifically, as shown in Figure 4f, the biexciton lifetimes for trinary, quaternary, and quinary metal NCs are nearly identical, irrespective of the NC size. On the contrary, the exciton lifetimes reach a minimum in the trinary NCs. Despite very small NC sizes for HEP NCs, the exciton lifetimes increase again upon HEA, consistent with the trend in exciton binding energy (Figure 3c). We therefore conclude that the exciton binding energy underpins the observed exciton kinetics.

### Computational Insight into the Electronic Structure

**Figure 5:**
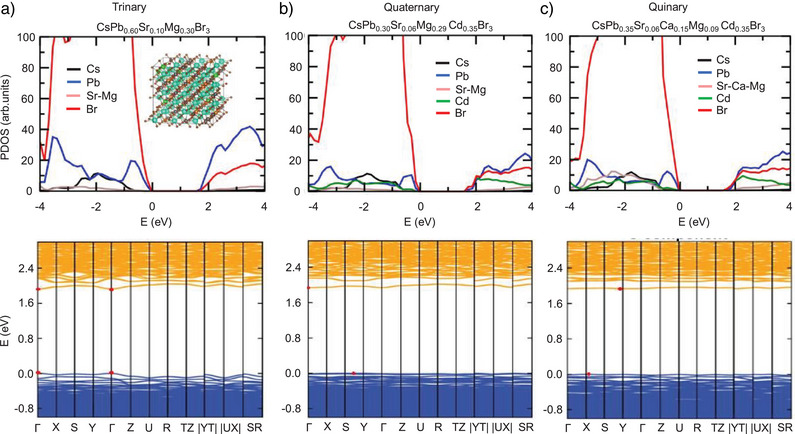
Calculated electronic band structures of HEP systems. DFT‐calculated projected density of states (PDOS; top row) and corresponding electronic band structures along high symmetry directions (bottom row) for representative a) trinary Cs(PbSrMg)Br_3_, b) quaternary Cs(PbSrCdMg)Br_3_, and c) quinary Cs(PbSrCaCdMg)Br_3_. The valence and conduction bands correspond to blue and orange curves, respectively, and red points highlight the band minima/maxima. The inset shows a representative trinary supercell in DFT calculations.

We carried out the DFT simulations to elucidate the electronic structures of HEPs. We considered representative trinary, quaternary, and quinary systems with B‐site elemental compositions given by the experimental characterizations of Cs(PbSrMg)Br_3_, Cs(PbSrCdMg)Br_3_, and Cs(PbSrCaCdMg)Br_3_ NCs. For tractable computations, we constructed special quasi‐random structures with 360‐atom supercells which approximate the disordered arrangement of B‐site atoms. Figure 5 presents the calculated electronic structures.^[^ ^81^ ^]^ The valence band maximum (VBM) in HEPs is predominantly composed of Br with some contribution from Pb, while the conduction band minimum (CBM) is primarily dominated by Pb with a smaller contribution from Br, resembling the CsPbBr_3_ system.^[^ ^82^ ^]^ In the four‐ and five‐component systems containing Cd, there is also significant Cd *p*‐like character at the CBM. We note that the Cd dominated CBM states are separated energetically from the continuous conduction bands by about 0.1 eV, which might correspond to shallow trap states observed in the TRPL responses. Although the alkaline earth metals (i.e., Sr, Ca, Mg) contribute marginally to the band edge states, they could potentially form MBr_2_ vacancy states that exist close to the band edge, as suggested by their lower vacancies formation energies compared to Cd (Figure S27).

For quaternary and quinary HEPs, the calculated band structures (Figure 5b, c) reveal that they are indirect band gap semiconductors with the VBM and CBM located away from Γ‐point. These HEP systems having a large degree of Pb replacement also exhibit relatively flat and non‐dispersive bands. In contrast, for the trinary system with a relatively higher Pb content, the direct band gap is located at the Γ‐point with a much more dispersive band edge states, similar to pure CsPbBr_3_ NCs.^[^ ^83^ ^]^ Figure S28a compares the DFT calculated bandgap values, suggesting that HEA results in a drastic bandgap widening. This increase in bandgap has been reported in other doped perovskite nanocrystals,^[^ ^84^ ^]^ which is attributed to the crystal symmetry breaking from the bonding and anti‐bonding orbitals in perovskites.^[^ ^85^ ^]^ Interestingly, as the degree of alloying increases, the calculated bandgap values remain approximately constant within the narrow energy range, in agreement with our experimental photophysical measurements.

We further extracted the electron and hole effective masses by parabolic fitting of the band edge states (Figure S28b) and calculated the Bohr radii using these effective masses together with dielectric constants obtained using Density Functional Perturbation Theory (Figure S28c), revealing a gradual increase of the effective masses with the decreasing Pb content. It underlies the increasingly non‐dispersive nature of electronic structure upon HEA, as the presence of Pb yields stronger covalent interactions in the system, leading to more dispersive bands. The increase of effective mass also contributes to a smaller excitonic radius, leading to faster radiative recombination rates and shorter radiative lifetimes,^[^ ^85^, ^86^ ^]^ consistent with our experimental observations, as illustrated in Figures 2, 3, 4.

### Electronic Band Dispersion in HEP NCS

**Figure 6:**
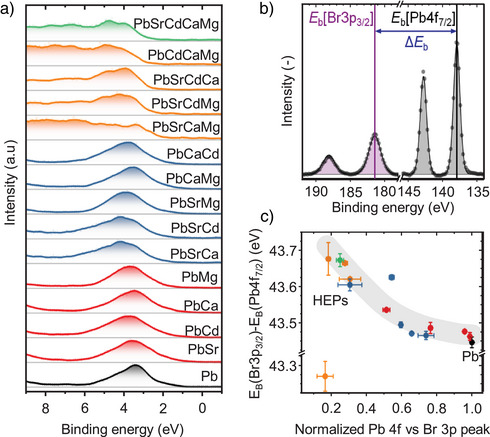
XPS characterization revealing electronic structures of HEP NCs. a) Low‐energy XPS spectra for parent CsPbBr_3_ (Pb) and all B‐site doped NC samples resolving the density of states at the conduction band edge. b) Concept of relative binding energies for robust semiconductor surface analysis. c) Correlation of Pb 4f peak area attenuation versus Br 3p reference against the relative binding energy.

To further elucidate the electronic structures of HEP NCs experimentally, we carried out extensive X‐ray photoelectron spectroscopy (XPS), which directly informs the density of states at the valence band edge (Figure 6a). While the parent CsPbBr_3_ NCs exhibits sharp valence states, we observed an increased dispersion for the valence band states upon alloying. In particular, for quaternary and quinary HEP NCs, the spectra reveal featureless and broad distribution of states, in agreement with the linear absorption, temperature‐dependent TRPL, and DFT simulation results discussed earlier. Analogous to previous findings in high‐entropy nanoparticles,^[^ ^87^ ^]^ the valence band dispersion results from the hybridization of states within the B‐site elements incorporated into the perovskite lattices. The findings nicely align with the observed extended Urbach tails in absorption spectra and rapid photoemission upon HEA (Figures 2d and S12). Intriguingly, our findings reveal that the formation of the “shallow bands” takes an advantage from the defect tolerance nature of LHP lattices, such that they do not serve as trapping centers/deep states for non‐radiative channels.

We further analyzed the chemical states extracted from the core level spectra. The Cs 3d, Br 3p, and Pb 4f features remain highly symmetric for each metal‐doped NC sample (Figures S29–S43), indicating the presence of a single dominant chemical state for each individual element. Notably, for XPS analysis of semiconductors and insulators, the charging effects may induce shifts in the spectra on the energy scale.^[^ ^88^ ^]^ Although charge referencing based on adventitious carbon is often applied to counter this effect, insufficient electrical contact of the adsorbate layer to the investigated sample could induce uncertainties of up to 2 eV.^[^ ^89^ ^]^ To circumvent these issues, internal charge referencing has recently been established as a technique to perform precise XPS analysis for semiconductors,^[^ ^90^, ^91^, ^92^, ^93^ ^]^ where the relative distance of two features inherent to the investigated samples are compared. Similar to our recently reported CsPbI_3_ nanoplatelets,^[^ ^94^ ^]^ we extracted the relative distance (Δ*E*_b_) between the Pb‐site (Pb 4f_7/2_) and halide‐site (Br 3p_5/2_) peaks to qualitatively analyze the local chemical environment of the Pb‐site, as schematically illustrated in Figure 5b.

Applying this concept on each sample considered here yields a clear correlation of the Pb chemical state in relation to its attenuation relative to the Br 3p peak (Figure 5c). With increasing alloying of the B‐site element, a continuous change of its chemical state is observed. The relative increase of the binding energies by up to 0.2 eV suggests an increased electron density at the Pb‐site relative to the Br reference. Strikingly, this trend is not governed by the type of metal incorporated into the lattice, despite their variable electronegativity. This suggests that the structural change induced upon alloying, such as the octahedral contraction previously observed from the structural analysis (Figure 2a, b), dominates change in the Pb─Br bonding. Interestingly, Cs(PbSrMg)Br_3_ and its quaternary derivative Cs(PbSrCaMg)Br_3_ exhibited a chemical state that did not fall into this overall trend. This may result from the formation of structural defects on the NC surface for these samples and/or the lower suitability of these dopants.

### Enhanced Photostability in HEP NCS

**Figure 7:**
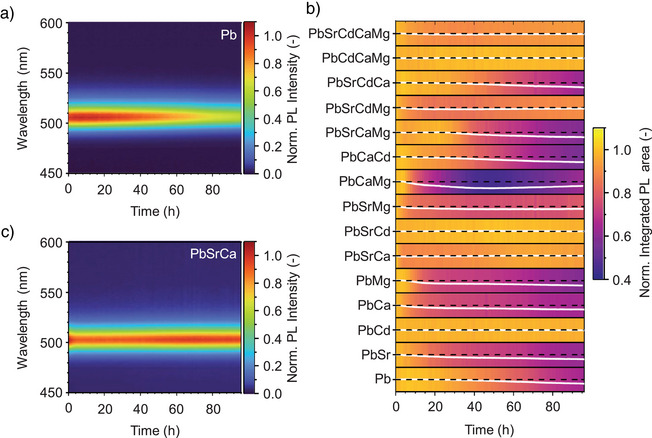
Enhanced photostability of metal‐alloyed perovskite NCs. Spectral stability of in situ PL aging under ambient conditions (25 °C, 60%–70% rel. humidity) for a) CsPbBr_3_ benchmark and b) trinary highly stable but reduced toxicity Cs(PbSrCa)Br_3_ samples, retaining 68% and 97% of their initial PL intensity, respectively, after 96 h of continuous automated test. c) Normalized integrated PL intensity with respect to time for all metal‐doped perovskite NC samples considered in this study.

Photostability of luminescent semiconductor nanocrystals has been recognized as the key consideration towards advanced optoelectronic applications.^[^ ^95^, ^96^ ^]^ We have examined the environmental stability of the HEP NCs considered here by in situ monitoring the thin‐film PL loss without any encapsulation (Figures 7 and S44). Taking advantage of an automated platform for optical stability assessment,^[^ ^97^ ^]^ we were able to resolve the often non‐linear changes with high temporal resolution over 96 h. As a benchmark, the CsPbBr_3_ thin‐film sample retained 68% of its initial PL intensity within the timeframe. Among the quaternary and quinary HEP NCs, the least stable sample exhibited similar performance with CsPbBr_3_, while the most stable sample substantially outperformed, preserving 95% of their initial PL intensity.

Interestingly, among the B‐site alloyed samples considered here, most of those exhibiting substantially enhanced photostability contain Cd^2+^, whereas Mg^2+^ alloying generally showed inferior performance. Indeed, Mg^2+^ alloying without Cd^2+^ may promote decomposition of the perovskite phase on the surface, because among all divalent metal ions considered here, Mg^2+^ has the smallest Shannon ionic radius (72 pm),^[^ ^98^ ^]^ hence with the largest difference compared to Pb^2+^ (119 pm).^[^ ^98^ ^]^ The ionic radius for Pb^2+^ is the most ideal for the stabilization CsPbBr_3_ perovskite phase according to the Goldschmidt criteria.^[^ ^99^ ^]^ On the contrary, the more similar ionic radius of Cd^2+^ (95 pm)^[^ ^98^ ^]^ might alleviate the shortcomings; for example in the Cs(PbCaCdMg)Br_3_ NC composition. The enhanced photostability of Cd^2+^‐incorporated NCs can be attributed to i) similar ionic radii for Cd^2+^ and Pb^2+^ and ii) the higher Pb‐like electronic contribution of Cd to the band‐edge states compared to other alkaline earth metal dopants, which promotes wavefunction hybridization. In addition, we also notice that the DFT‐calculated defect formation energy for CdBr_2_ vacancies is considerably higher than other divalent metal ion bromide vacancies (Figure S27).

Remarkably, the systematic experimentation allowed us to identify a trinary B‐site alloyed NCs compound, Cs(PbSrCa)Br_3_, showing extraordinary photostability with reduced toxicity by retaining (97 ± 2)% of its initial PL intensity within 96‐hour timeframe (Figure 7c). We consider this compound particularly attractive for commercial optoelectronic applications. The similar ionic radii of Sr^2+^ (118 pm) and Ca^2+^ (100 pm) appear even more suitable for B‐site alloying as compared to Cd^2+^, but the binary Cs(PbSr)Br_3_ and Cs(PbCa)Br_3_ only showed similar stability with parent CsPbBr_3_ NCs, retaining (61 ± 3)% and (66.1 ± 0.3)% of their initial PL intensities, respectively. This underscores that HEA enhances the PL stability owing to the different ionic radii of Sr^2+^ and Ca^2+^, which generates local strains by the formation of lattice expansion and interstitial phases.^[^ ^39^ ^]^ We also notice that by introducing Sr^2+^, the relatively low photostability of Cs(PbMg)Br_3_ (59.6 ± 0.2%) was substantially enhanced (77.8 ± 0.6%) for Cs(PbSrMg)Br_3_. Consequently, the HEP NCs generally exhibit enhanced stability compared to binary‐alloyed NCs. Nevertheless, we observed reduced photostability in HEP NCs compared to the trinary species, which could be attributed to the introduction of excessive defects during multicationic exchange, as suggested by their lower solution *η*_PL_ and exciton binding energy (Figure 3a, c). The complex interplay between different B‐site alloying elements motivates data‐driven compositional optimization and further in‐depth investigations to elucidate their synthesis‐structure‐performance relationships.

## Conclusion

In summary, we have synthesized a series of HEP NCs based on parent CsPbBr_3_ perovskite system. The HEP NCs exhibit both enhanced *η*_PL_ and shortened PL lifetimes. Although the HEP NCs have lateral size smaller than the Bohr diameter of parent CsPbBr_3_ NCs, their absorption spectra show the absence of confined resonance and the emergence of long‐tail states, which cannot be explained using the classical quantum theory. Detailed temperature‐dependent photophysical analyses reveal that B‐site metal doping could increase the exciton binding energy and introduce shallow states near the band edge. The shallow states synergistically facilitate electron‐hole radiative recombination with intrinsic band‐edge photon emission. Our DFT simulations suggest that HEA reduces the band dispersion, thereby resulting in higher effective electron and hole masses. The in‐depth XPS measurement endorses the presence of shallow states and discloses gradual broadening of valence states with increasing degree of B‐site alloying. Moreover, by monitoring the in situ PL intensity change, we identified the HEP NC thin films with substantially enhanced photostability and reduced toxicity. Our findings uncover the fundamental mechanisms altering photophysical properties of HEP NCs, bridging the gap to correlate the electronic structure and the alloying degree. We believe that the data‐driven optimization of compositional space for the development of next‐generation luminescent QDs will be greatly facilitated by the fundamental principles and chemical methodology presented here.

## Supporting Information

Additional supporting information can be found online in the Supporting Information section at the end of this article.

## Author Contributions

C.J.S. conceived the idea and coordinated the project; Y.T.C., S.B.S., and A.W. performed the majority of experiments and analyzed the data with input from S.K, S.S, Y.‐T.L, Y.‐C.C., and C.J.S. Z.L. generated the SQS, and K.H.K. and Y.L. performed and supervised the DFT calculations, respectively. J.W.M.L. performed the TA experiments and analyzed data with Z.X., supervised by T.C.S. Y.T.C. and S.B.S. co‐wrote the manuscript with input from A.W., Z.X., Y.L., S.K., S.F.S., and C.J.S. All authors commented on and approved the manuscript.

## Conflict of Interests

The authors declare no conflict of interest.

## Supporting information



Supporting information

## Data Availability

The data that support the findings of this study are available from the corresponding author upon reasonable request.
