# Supplementary material for: Understanding Optical Properties and Electronic Structures of High‐Entropy Alloyed Perovskite Nanocrystals
Source: Angew Chem Int Ed Engl. 2025 Jul 24;64(37):e202505890. doi: 10.1002/anie.202505890 (PMC12416448; doi:10.1002/anie.202505890)
Supplement: Supplementary file 1 — Supporting information [file ANIE-64-e202505890-s001.docx]

**Supporting Information**

**"Understanding Optical Properties and Electronic Structures of High-Entropy Alloyed Perovskite Nanocrystals"**

*Yung-Tai Chiang*^1, #^, *Sunil B. Shivarudraiah*^1, #^, *Alexander Wieczorek*^2^, *Khoong Hong Khoo*^3^,

*Zhidong Leong*^3^, *Jia Wei Melvin Lim*^4^, *Zengshan Xing*^4^, *Sudhir Kumar*^1^, *Simon F. Solari*^1^, *Yen-Ting Li*^5,6^*, Yu-Cheng Chiu*^5,7^, *Tze Chien Sum*^4^*, *Yun Liu*^3^*, *Sebastian Siol*^2^*, *Chih-Jen Shih*^1^*

1. Institute for Chemical and Bioengineering, ETH Zürich, 8093 Zürich, Switzerland
2. Laboratory for Surface Science and Coating Technologies, Empa – Swiss Federal Laboratories for Materials Science and Technology, 8600 Dübendorf, Switzerland
3. Institute of High Performance Computing (IHPC), Agency for Science, Technology and Research (A*STAR),1 Fusionopolis Way, 16-16 Connexis, Singapore 138632, Republic of Singapore
4. Division of Physics and Applied Physics, School of Physical and Mathematical Sciences, Nanyang Technological University, 21 Nanyang Link, Singapore 637371, Singapore
5. Department of Chemical Engineering, National Taiwan University of Science and Technology, Taipei 10607, Taiwan
6. National Synchrotron Radiation Research Center, Hsinchu 30076, Taiwan
7. Advanced Research Center for Green Materials Science and Technology, National Taiwan University, Taipei 10617, Taiwan

# These authors contribute equally.

* To whom correspondence should be addressed: [tzechien@ntu.edu.sg](mailto:tzechien@ntu.edu.sg); [liu_yun@ihpc.a-star.edu.sg](mailto:liu_yun@ihpc.a-star.edu.sg); [sebastian.siol@empa.ch](mailto:sebastian.siol@empa.ch); [chih-jen.shih@chem.ethz.ch](mailto:chih-jen.shih@chem.ethz.ch)

1. **Supplementary methods**

**1.1 Chemicals**

Toluene (99.8%, Fisher Chemical), oleic acid (OLA, 90%, technical grade), oleylamine (OLAM, approximate C18-content 80–90%, Acros Organics), 1-octadecene (ODE, Thermo scientific - Acros Organics), lead (II) bromide (PbBr_2_, 98+%, Thermo scientific - Acros Organics), cesium carbonate (Cs_2_CO_3,_ 99.5%, Thermo scientific - Acros Organics), tetraoctylammonium bromide (TOAB, 98%, Fluorochem), magnesium bromide anhydrous (MgBr_2_, 98%, anhydrous, ABCR GmbH), strontium bromide anhydrous (SrBr_2_, 98%, Thermo scientific - Acros Organics), cadmium bromide anhydrous (CdBr_2_, 99%, Strem Chemicals), calcium bromide (CaBr_2_, 99%, anhydrous, Thermo scientific - Acros Organics), methyl acetate (99%, ABCR GmbH), ethyl acetate (99.9% extra dry over molecular sieve, Thermo scientific - Acros Organics), acetonitrile anhydrous (99.8%, Sigma-Aldrich), lecithin from soybean (Tokyo Chemical Industry), zinc bromide anhydrous (ZnBr_2_, 99%, Thermo scientific - Acros Organics). All chemicals listed above were used as received without further purification

**1.2 Synthesis of pristine CsPbBr_3_ NCs and HEP NCs**

*Cesium Oleate Precursor Solution*. Cs_2_CO_3_ (1 mmol) was dissolved in OLA (10 mL). The precursor solution was stirred until Cs_2_CO_3_ powder was completely dissolved.

*Synthesis of CsPbBr_3_ NCs:* PbBr_2_ (367 mg) and TOAB (1093 mg) were loaded in a 100mL round-bottomed one-neck flask, and 10mL of toluene was added into the flask to dissolve PbBr_2_ and TOAB. Note that here TOAB only assists in the dissolution of PbBr_2_ in toluene, and it will not act as ligands binding to the surface of NCs. To trigger the precipitation of NCs, 1.15mL Cs-oleate precursor was swiftly injected into the round-bottomed one-neck flask under vigorous stirring, and the solution turned from clear to turbid rapidly (~3s), indicating the rapid formation of NCs. The reaction was stirred at room temperature in open air for 5 minutes. Subsequently, 23.1μL OLAM was added into the reaction solution.

*Remove of Excessive Ligands by Washing.* After 2 minutes, 3mL acetonitrile was added into the crude solution to remove the excessive ligands. The precipitation was collected by centrifugation (8,000 rpm, 8 minutes) and redispersed in toluene (8mL). The CsPbBr_3_ NCs suspension was then filtered through a syringe filter (pore size 0.2μm). The concentration of the CsPbBr_3_ NCs is estimated to be ~1.2 mg/mL.

Note: All post-synthetic cation exchange reactions are conducted inside the glovebox using a modified protocol from the previous report.^1^

*Synthesis of binary alloyed CsPbBr_3_ NCs (i.e. Cs(PbSr)Br_3_, Cs(PbCa)Br_3_, Cs(PbCd)Br_3_, and Cs(PbSrMg)Br_3_)*

Single alloyed CsPbBr_3_ NCs was prepared by ligand-assisted cationic exchange at room temperature. In the first step, metal bromide salt (MBr_2_, with M = Sr, Ca, Cd, Mg) is partially dissolved with 10mg Lecithin, 10μL OLAM, and 490μL toluene in a 10mL vial and shortly stirred. The amount of SrBr_2_, CaBr_2_, CdBr_2_, and MgBr_2_ is 62mg (0.25mmol), 50mg (0.25 mmol), 17mg (0.063mmol), and 12mg (0.063mmol), respectively. Subsequently, 1.5mL pristine CsPbBr_3_ NCs stock solution was added into the prepared metal bromide solution and the reaction solution was stirred vigorously for 16hrs. The reaction mixture was then separated by centrifugation (5,000rpm, 8 minutes), and the single alloyed CsPbBr_3_ NCs suspension was filtered through a syringe filter (pore size 0.2μm). Different single alloyed CsPbBr_3_ NCs suspension were then washed by different anti-solvents via centrifugation to remove unreacted metal bromide salts (10,000rpm, 5 minutes). Specifically, the Cs(PbSr*)*Br_3_ was washed with 400μL mixture of acetonitrile and methyl acetate (volume ratio 1:1). The Cs(PbCa)Br_3_ and Cs(PbMg)Br_3_ were washed with 600μL methyl acetate. The Cs(PbCd)Br_3_ was washed with 600μL ethyl acetate. After centrifugation, the supernatant was discarded and the purified single alloyed CsPbBr_3_ NCs were redispersed in toluene for further characterization.

*Synthesis of trinary alloyed CsPbBr_3_ NCs (i.e Cs(PbSrCa)Br_3_, Cs(PbSrCd)Br_3_, Cs(PbSrMg)Br_3_, Cs(PbCMg)Br_3_, Cs(PbCaCd)Br_3_, and Cs(PbCdMg)Br_3_)*

In a typical synthesis of binary alloyed CsPbBr_3_, two metal bromide salts (M_A_Br_2_ and M_B_Br_2_ with M_A_, M_B_ = Sr, Ca, Cd, Mg and M_A_ ≠ M_B_) are partially dissolved together with 10mg Lecithin, 10μL OLAM, and 490μL toluene in a 10mL vial and shortly stirred. The amount of SrBr_2_, CaBr_2_, CdBr_2_, and MgBr_2_ is 0.25mmol, 0.25 mmol, 0.063mmol, and 0.063mmol, respectively. Subsequently, 1.5mL pristine CsPbBr_3_ NCs stock solution was added into the metal bromide solution and the reaction solution was stirred vigorously for 16hrs. The reaction mixture was then separated by centrifugation (5,000rpm, 8 minutes) and the binary alloyed CsPbBr_3_ NCs suspension was filtered through a syringe filter (pore size 0.2μm). Different binary alloyed CsPbBr_3_ NCs suspension was washed by different anti-solvents under centrifugation to remove unreacted metal bromide salts (10,000rpm, 5 minutes). Specifically, the Cs(PbSrCa)Br_3_ NCs was washed with 600μL methyl acetate; Cs(PbSrMg)Br_3_ and Cs(PbCaMg)Br_3_ NCs are washed with 600μL mixture of ethyl acetate and methyl acetate (volume ratio 1:1); Cs(PbSrCd)Br_3_, Cs(PbCaCd)Br_3_, and Cs(PbCdMg)Br_3_ NCs are washed with 600μL ethyl acetate. After centrifugation, the supernatant was discarded and the purified binary alloyed CsPbBr_3_ NCs were redispersed in toluene for further characterization.

*Synthesis of high-entropy alloyed (quaternary and quinary alloyed) CsPbBr_3_ NCs (i.e. Cs(PbSrCaMg)Br_3_, Cs(PbSrCdMg)Br_3_, Cs(PbSrCaCd)Br_3_, Cs(PbCaCdMg)Br_3_, and Cs(PbSrCaCdMg)Br_3_)*

High-entropy alloyed CsPbBr_3_ NCs were prepared by multi-cationic exchange. Three or four metal bromide salts (M_A_Br_2_, M_B_Br_2_, M_C_Br_2_, and M_D_Br_2_ with M_A_, M_B_, M_C_, M_D_ = Sr, Ca, Cd, Mg and M_A_ ≠ M_B_ ≠ M_C_ ≠ M_D_) are partially dissolved together with 10mg Lecithin, 10μL OLAM, and 490μL toluene in a 10mL vial and shortly stirred. The amount of SrBr_2_, CaBr_2_, CdBr_2_, and MgBr_2_ is 0.125 mmol, 0.125 mmol, 0.031 mmol, and 0.031 mmol, respectively. Subsequently, 1.5mL pristine CsPbBr_3_ NCs stock solution was added into the prepared metal bromide solution and the reaction solution was stirred vigorously for 16hrs. The reaction mixture was then separated by centrifugation (5,000rpm, 8 minutes) and the high-entropy alloyed CsPbBr_3_ NCs suspension was filtered through a syringe filter (pore size 0.2μm). We did not washed the high-entropy CsPbBr_3_ NCs due to deterioration after washing and the difficulty for precipitation.

*Synthesis of single, binary, and high-entropy alloyed quantum-confined CsPbBr_3_ nanocrystals (QC CsPbBr_3_ NCs)*

We modified a method based on Boehme *et al.* to synthesize quantum confined CsPbBr_3_ NCs.^2^

*Cesium Oleate 0.16 M in Octadecene (ODE)*. 0.77 mmol Cs_2_CO_3_ was mixed with oleic acid (0.8 mL) and ODE (8.8 mL) in a 50 mL three neck flask. The mixture was degassed for one hour and then heated to 120 °C under N_2_ until it became clear. Cesium oleate in ODE was stored in the under N_2_.

*Synthesis of Quantum-confined CsPbBr_3_ Nanocrystals.* PbBr_2_ (300mg) and ZnBr_2_ (720 mg) were mixed together within 20mL of ODE in a 50 mL three-neck flask under N_2_. The mixture was then heated to 100 °C and degassed for one hour. Distilled oleylamine (8 mL) and dried oleic acid (8 mL) were slowly injected. The mixture was heated to 145 °C in order to dissolve all the metal bromide powder. 1.6 mL cesium oleate was swiftly injected from a 2mL glass syringe. The reaction was quenched after 15 s with ice bath.

*Size selective washing.* The crude solution was centrifuged (10,000 rpm, 5 min), and the precipitate was discarded. Ethyl acetate (1:3 volume ratio) was added to the supernatant, and then it was centrifuged (10,000 rpm, 5 min). The supernatant was the discarded, and the precipitate was dispersed in 1.5 mL toluene.

**1.3 Characterisation**

***Photoluminescence quantum yield (PLQY) spectroscopy***

PLQY characterizations of the liquid and thin film samples were performed by using a Hamamatsu Quantaurus QY absolute η_PL_ spectrometer (C11347-11) equipped with a 150 W xenon lamp and a 3.3 inch integrating sphere, which is coated with highly reflective Spectralon. The PL spectra were measured at an excitation wavelength of 370 nm. The liquid samples were diluted up to 200 times with toluene. The thin film samples were prepared by dripping 50μL of the NCs suspension on a 16mm × 16mm glass chip and dried patiently.

***Time-resolved photoluminescence (TRPL) spectroscopy***

TRPL spectra of the NCs dispersions were acquired using a Hamamatsu Quantaurus-Tau Fluorescence Lifetime Spectrometer (C11367-31), which is equipped with a photon counting measurement system. The excitation wavelength was set at 365 nm and the measurements were recorded using a pulse repetition rate of 500 kHz. The liquid samples were diluted up to 200 times with toluene. The instrument response function (IRF) was measured using a LUDOX® AS-30 colloidal silica suspension (30 wt. % suspension in H_2_O, Aldrich) diluted with deionized water.

In order to carry out temperature dependent TRPL and PL intensity measurements for thin film, an Oxford Optistat DN cryostat was integrated with the Quantaurus-Tau. The samples were prepared by dripping 25 μL of the NCs suspension on 16mm × 16 mm glass substrates and dried patiently. For temperature dependent TRPL measurement, in order to resolve a better IRF, a faster laser with an excitation wavelength of 405 nm and a FWHM of ~0.4 ns was employed. All the TRPL data were fitted with a single-, bi-, or triple- exponential function. The change of spectrum bandwidth with respect to different temperatures was fitted by Frohlich equation: $Г\left( T \right)= Г_{0}+ Г_{LO}/(e^{\frac{E_{LO}}{k_{B}T}}-1)$, where the $Г_{0}$ is the broadening from lattice imperfection, $Г_{LO}$ is the exciton-phonon coupling strength, k_B_ the Boltzmann constant, and *E_Lo_* is the energy representative for the frequency of weakly dispersive phonon.

***UV-visible absorption spectroscopy***

UV-visible absorption spectra of the colloidal NCs were measured using a Cary 60 UV-Vis spectrophotometer from Agilent Technologies or a Jasco V770 spectrophotometer. The liquid samples were diluted 200 times with toluene. The scanning rate was 200nm/min.

***Transmission electron microscopy (TEM)***

TEM morphology analysis was performed by JEOL JEM-1400+ transmission electron microscope, which is equipped with LaB_6_ crystal filament emitter and JEOL CCD camera Ruby (8M pixel). The microscope was operated at 200kV. The samples were prepared by dripping a diluted NCs solution on a carbon-coated polymer film copper grid. Toluene is allowed to evaporate slowly before TEM measurement.

***Scanning electron microscope and energy-dispersive X-ray spectroscopy (SEM-EDS):***

SEM-EDS measurement was conducted using Zeiss ULTRA 55 scanning electron microscope equipped with a Xplore energy-dispersive spectroscopy detector. The samples were prepared by dripping 50μL NCs solution on Si/SiO_2_ (285 nm ± 5%) substrates. The applied voltage was 10kV. The collection time for a single measurement was 5 minutes and the dead time was maintained to be ~30%. Aztech Software was utilized to analyze the atomic percentage of different elements.

***High resolution transmission electron microscopy (HRTEM)***

High-resolution high-angle annular dark field scanning transmission electron microscopy (HAADF-STEM) measurements were performed using a TFS Talos F200X equipped with a field-emission gun operated at 200 kV. Cryo-conditions were used with a low background double tilt (maximum tilt angle ±30°) holder cooled using liquid nitrogen.

EDS measurements were performed using the same FEI Talos F200X under cryo-conditions with Super-​X EDS 4 detector configuration. All images (1,024 × 1,024 pixels) were acquired with a dwell time of 10 µs per pixel. The acquisition time for EDS measurements was ∼300 s.

Samples for imaging were prepared by dripping a 10 µl diluted nanocrystal solution in toluene on a formvar carbon film supported by a copper carrier grid (200 mesh). The solvent (toluene) was completely evaporated before loading into TEM. The grids were purchased from PLANO.

***X-ray diffraction (XRD)***

XRD was conducted using a Rigaku SmartLab diffractometer with a D/teX Ultra 250 detector using Cu Kα radiation (λ = 0.1541 nm) and operating in Bragg-Brentano geometry. The samples were prepared by drop casting 50μL concentrated pristine and alloyed CsPbBr_3_ NCs on a 10mm x 10mm Si/SiO_2_ (285 nm ± 5%) substrates. Data were acquired in the 5-70° 2θ range with an angular step size of 0.025° and a counting time of 3 s per step.

***Synchrotron grazing-incidence wide-angle X-ray scattering (GIWAXS) and small-angle X-ray scattering (GISAXS)***

Synchrotron GIWAXS and GISAX were conducted at the Taiwan Photon Source beamline BL13A at the National Synchrotron Radiation Research Center of Taiwan. The incidence angle and beam energy of the X-ray were 0.12° and 12.16 keV, corresponding to a wavelength of 1.02143 Å. The MAR165 CCD with a 2D area detector was used to collect all of the GIWAXS images in reflection mode.

***X-ray photoelectron spectroscopy (XPS)***

X-ray photoelectron spectroscopy (XPS) was performed in a PHI Quantera system at Empa (Swiss Federal Laboratories for Materials Science and Technology), in which samples were mounted on insulating substrates in order to minimize vertical differential charging effects. XPS measurements were performed at a pressure of 10^−9^ − 10^−8^ Torr. The monochromatic Al Kα radiation was generated from an electron beam at a power of 25.3 W and a voltage of 15 kV. To minimize beam damage during measurements, the beam spot with a diameter of 100 µm was continuously scanned over an area of 500 × 1,000 µm^2^. Charge neutralization was performed using a low-energy electron source. Short-term measurements (<1 min) of the Pb 4f core level before and after each presented long-term measurement (~30 min) were conducted to rule out changes in the chemical state due to X-ray induced beam damage. Peak fitting of photoelectron features was performed in Casa XPS using Voigt profiles with GL ratios of 60 following Shirley-background subtraction. The binding energy scale was referenced to the main component of adventitious carbon at 284.8 eV, resulting in a typical inaccuracy of ±0.2 eV. However, this inaccuracy is cancelled out for relative energy values, *e.g.* those between Pb 4f_7/2_ and Br 3p_3/2_ regions as performed here. For each measured composition, two areas on the same sample were measured to assess materials variations across the sample. Resulting measurement values were then averaged and the error Δ*E* for relative energy values was calculated according to:

$$\begin{aligned} \Delta E= \pm\frac{E_{2}-E_{1}}{2} \#\left( 1 \right) \end{aligned}$$

,where *E*_1_ and *E*_2_ denote the measured relative energy values for the areas 1 and 2 on each measured sample. Since quantifications of core-shell structures as found in perovskite nanocrystals may result in systemic errors, the quantification results were first normalized for CsPbBr_3_ and then used for the alloyed members as following: The stoichiometric coefficient for Cs and Pb in Cs*_m_*Pb*_n_*Br_3_ were first normalized to *m* = 1 and *n* = 1 for the parent CsPbX_3_ based on the Cs 3d, Pb 4f and Br 3p regions. For the alloyed members, the stoichiometric factors were then calculated based on the relative loss of intensity of the Cs 3d and Pb 4f integrated peak area compared to Br 3p.

***In-situ photoluminescence photostability measurements***

*In-situ* photoluminescence (PL) measurements were conducted using a custom-built setup for high-throughput optical measurements at Empa, as reported elsewhere.^3^ HEA NCs dispersions were first drop-casted on borosilicate glass substrates and quickly dried using a nitrogen (N_2_) flow. Two measurement spots were defined per sample composition. Repeated PL measurements were performed by rastering across all defined spots within approximately 1 h intervals under ambient conditions (25 °C, 60 – 70 % rel. humidity). Excitation was provided by a deuterium lamp source (Ocean Optics DH-2000-BAL) coupled to a collimating lens (Ocean Insight 74-ACR) *via* an optical fiber, yielding an output power of 0.2 mW.

***Transient absorption spectroscopy***

The pump-probe Transient Absorption (TA) measurements were performed using the commercial HELIOSTM femtosecond transient absorption spectrometer (Ultrafast Systems). The pump pulse at 400 nm is generated from optical parametric amplifier (OPerA Solo) powered by the regeneration amplifier (Coherent Libra, 800 nm, 1 kHz, 50 fs). To obtain the probe pulse (430-750 nm), a portion of the 800 nm fundamental beam was focused on a sapphire crystal to generate the supercontinuum which then passed through a 750 short pass filter to remove the fundamental beam. The pump and probe beams were overlapped on the sample, and the pump beam was chopped at 500 Hz to provide pump-on and pump-off states. The transmitted probe spectra were detected by the spectrometer equipped with a CMOS sensor.

***Density Functional Theory Calculation***

We aim to investigate the optical properties of high-entropy CsXBr_3_ perovskite materials by performing density functional theory (DFT) calculations using the VASP package that treats core electrons with the projector-augmented wave (PAW) method.^4^ Wavefunctions are represented using plane-waves with an energy cutoff of 280 eV and the exchange-correlation potential is treated using the Perdew-Burke-Ernzerhof (PBE) parametrization of the Generalized-Gradient Approximation.^5^ The Brillouin zone is sampled using the Monkhorst-Pack scheme with a grid spacing of 0.15 Å⁻¹.^6^ The lattice parameters were relaxed until the diagonal components of the stress tensor are below 10^-2^ GPa, and the crystal structure constrained in orthorhombic phase in agreement with experimental observation. The atomic positions were also relaxed until Hellman-Feynman force converges below 0.02 eV/Å. Since our system contains heavy elements such as Pb, we account for relativistic effects by including spin-orbit interactions in all electronic structure calculations.

**Special Quasi-random Structures (SQS)**

The SQS of high-entropy CsXBr_3_ perovskites are periodic structures with atoms on the B sites arranged such that the correlation functions of various elemental species approximate the expected values in an ideal infinite random arrangement. Using a simulated annealing approach, we optimised the B-site atomic arrangements with respect to correlation functions up to the eighth nearest neighbour pairs in the Pnma space group. The definitions of the correlation functions follow our previous cluster expansion formalism.^7^ Multiple initial random seeds were used in the optimisation algorithm to generate different SQS at the same composition and supercell size.

The SQS supercells are still too large for hybrid functionals plus spin-orbit interactions, which are needed to accurately describe the electronic structures of hybrid perovskites.^8, 9^ We tested the accuracy of semilocal functional PBE against hybrid functional HSE06^7^ (See SI). Our tests show that both methods yield similar qualitative features in the projected density of states (PDOS), with the differences between PBE and HSE06 calculations well-represented by a simple scissor shift. We therefore use PBE with spin-orbit interactions for electronic structure calculations.

**Supplementary Figures and Tables**


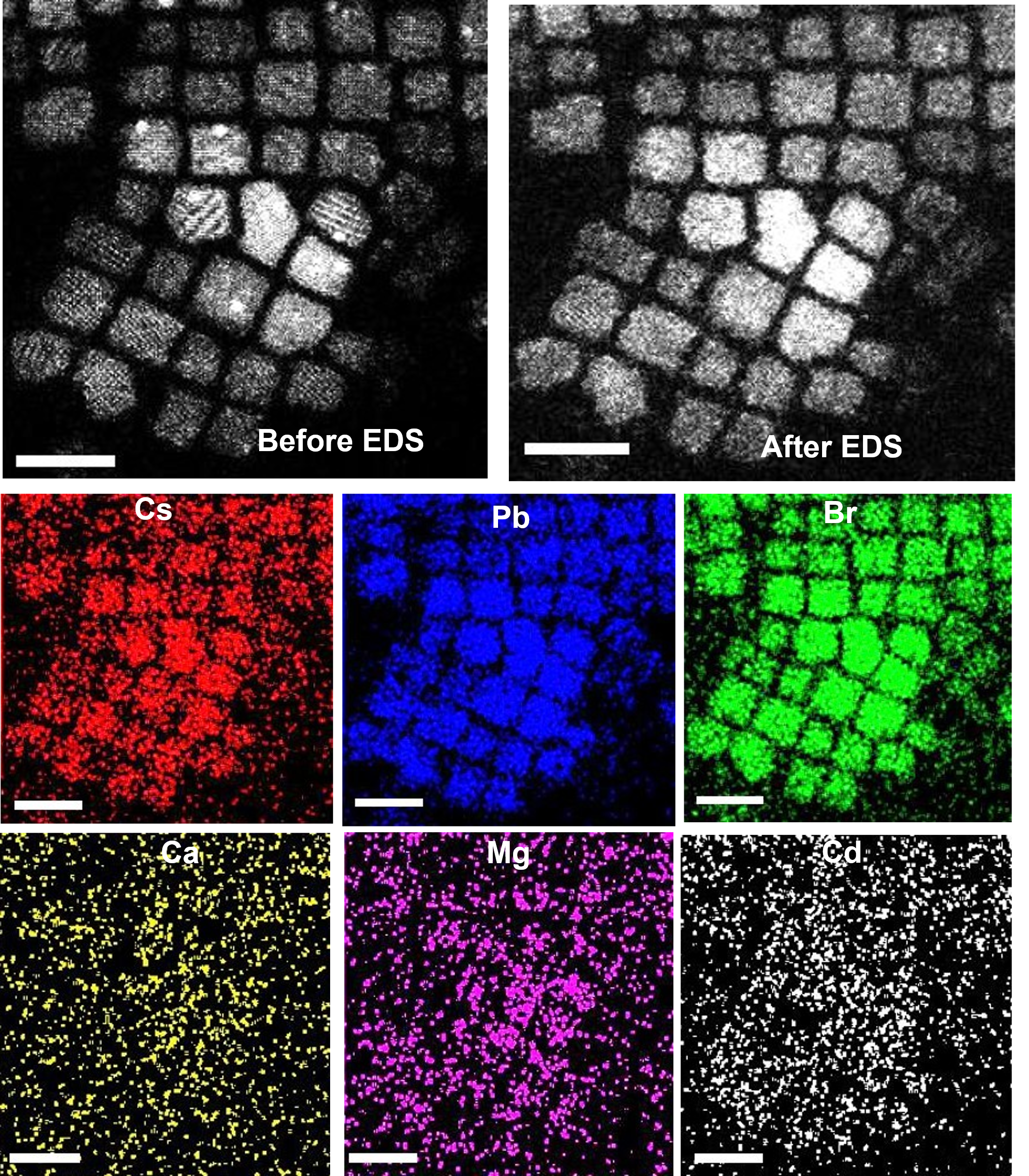


**Figure S1.** HAADF-STEM image of Cs(PbCaCdMg)Br_3_ NCs and the corresponding elemental maps, demonstrating the presence of Ca, Cd, Mg.


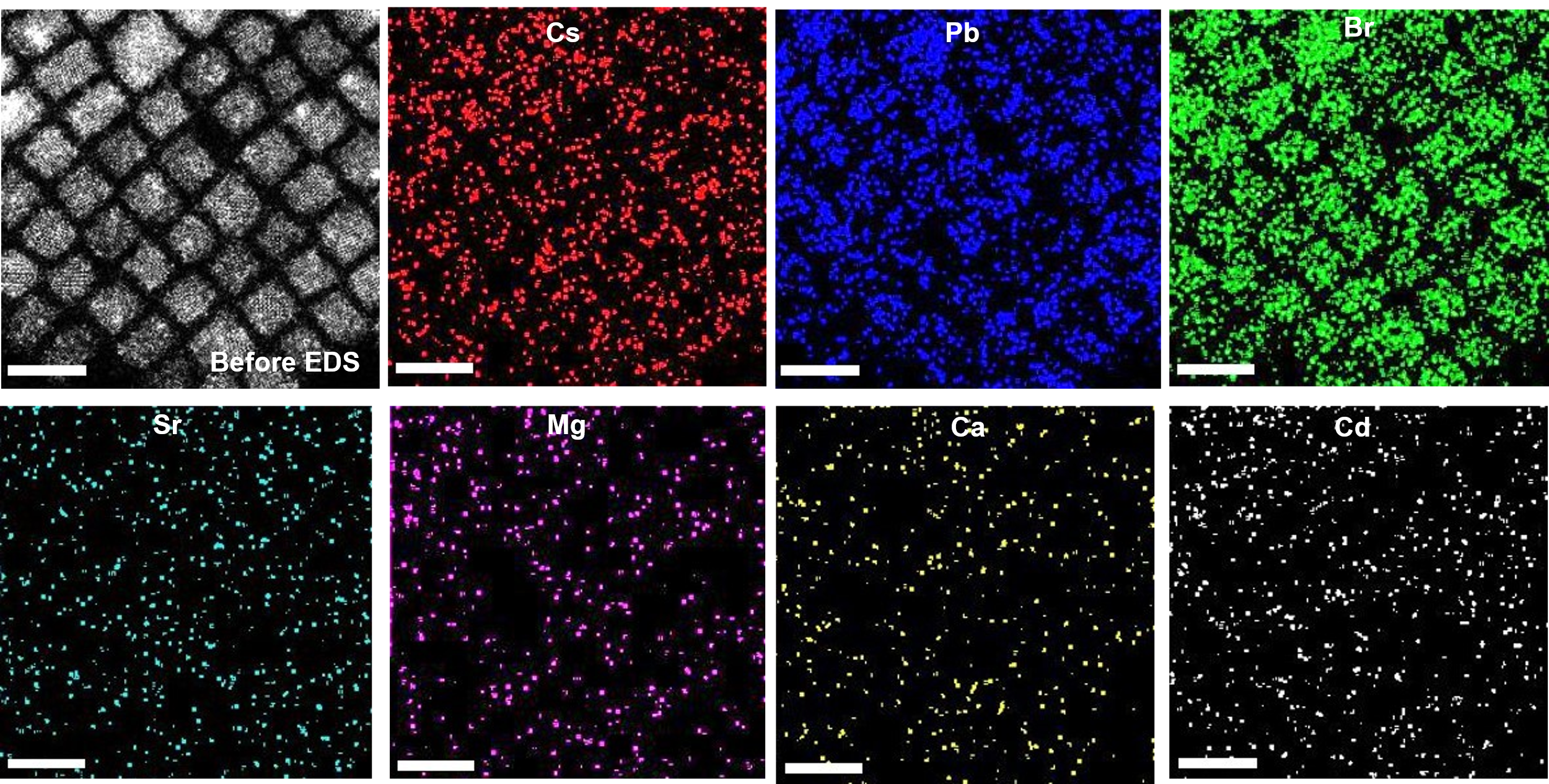


**Figure S2.** HAADF-STEM image of Cs(PbSrCaCdMg)Br_3_ NCs and the corresponding elemental maps, demonstrating the presence of Sr, Ca, Cd, Mg.


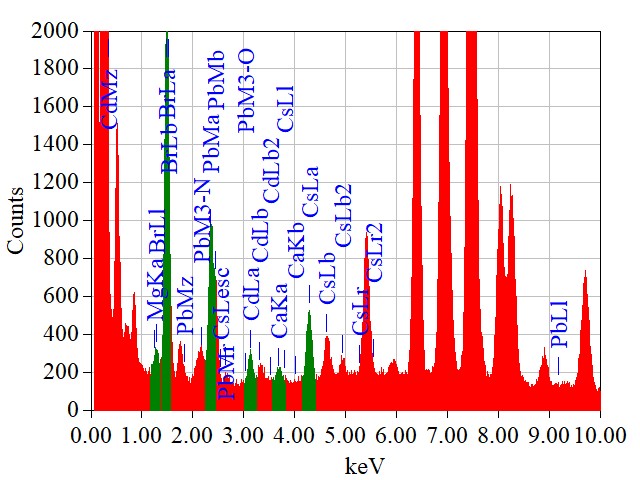


**Figure S3**. Energy Dispersive X-ray Spectroscopy of Cs(PbCaCdMg)Br_3_ NCs.


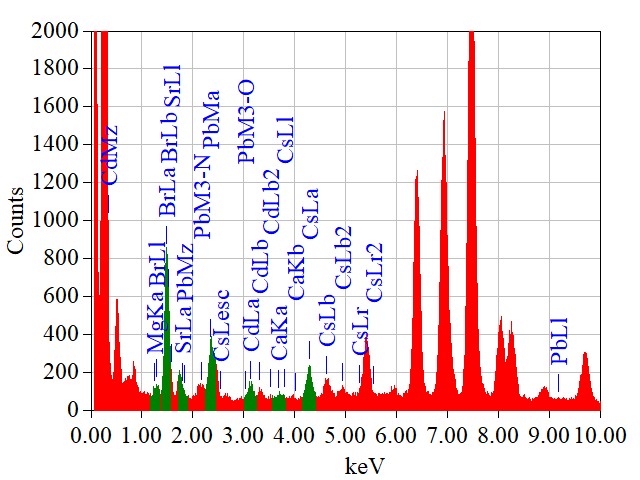


**Figure S4**. Energy Dispersive X-ray Spectroscopy of Cs(PbSrCaCdMg)Br_3_ NCs.


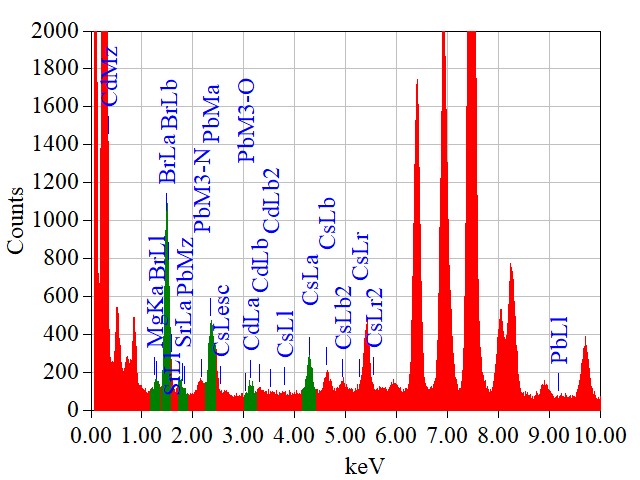


**Figure S5**. Energy Dispersive X-ray Spectroscopy of Cs(PbSrMgCd)Br_3_ NCs.


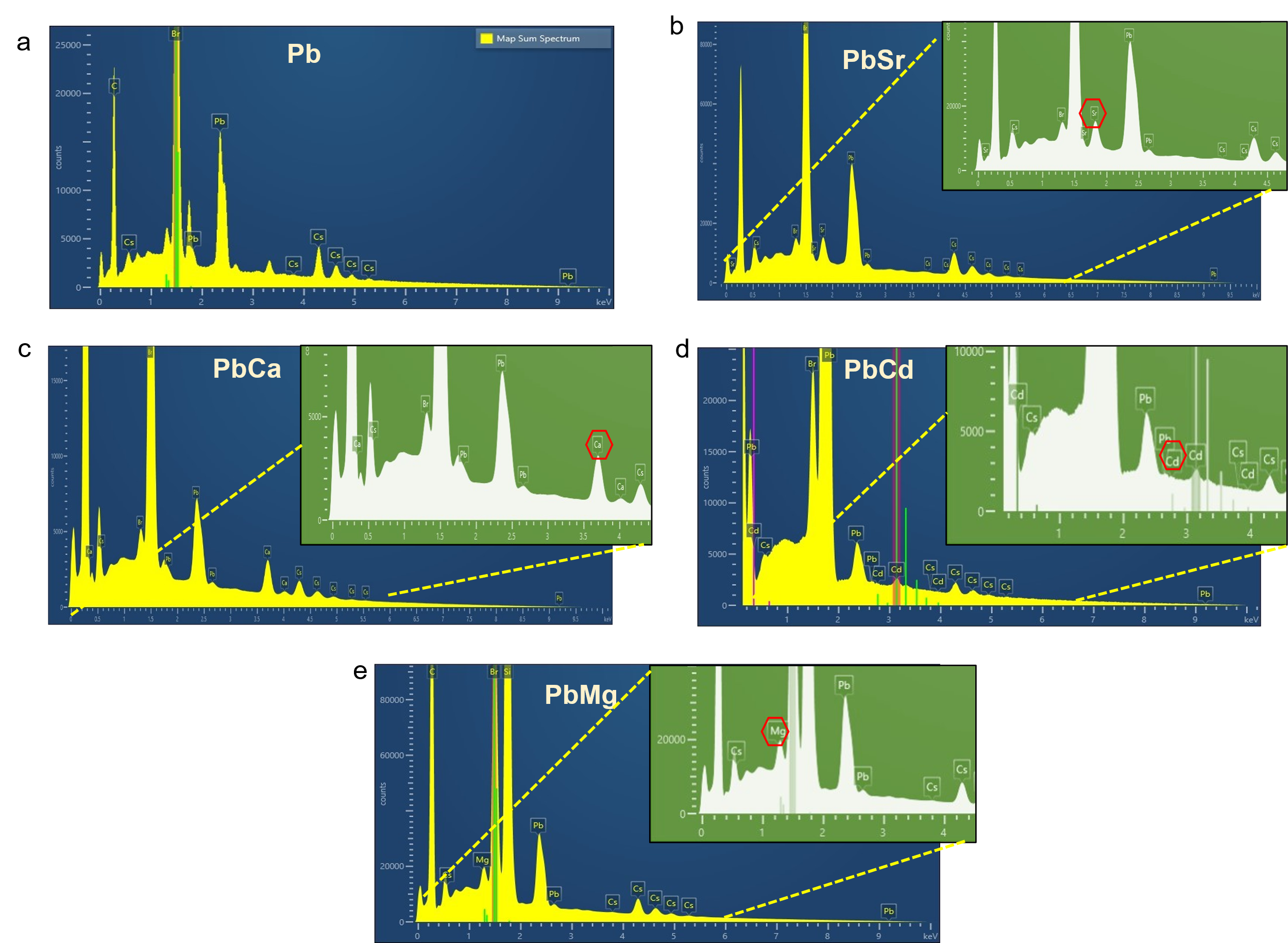


**Figure S6**. The SEM EDS spectrum for parent and binary alloyed NCs. a) CsPbBr_3_ NCs. b) Cs(SrPb)Br_3_ NCs. c) Cs(PbCa)Br_3_ NCs. d) Cs(PbCd)Br_3_ NCs. e) Cs(PbMg)Br_3_ NCs.


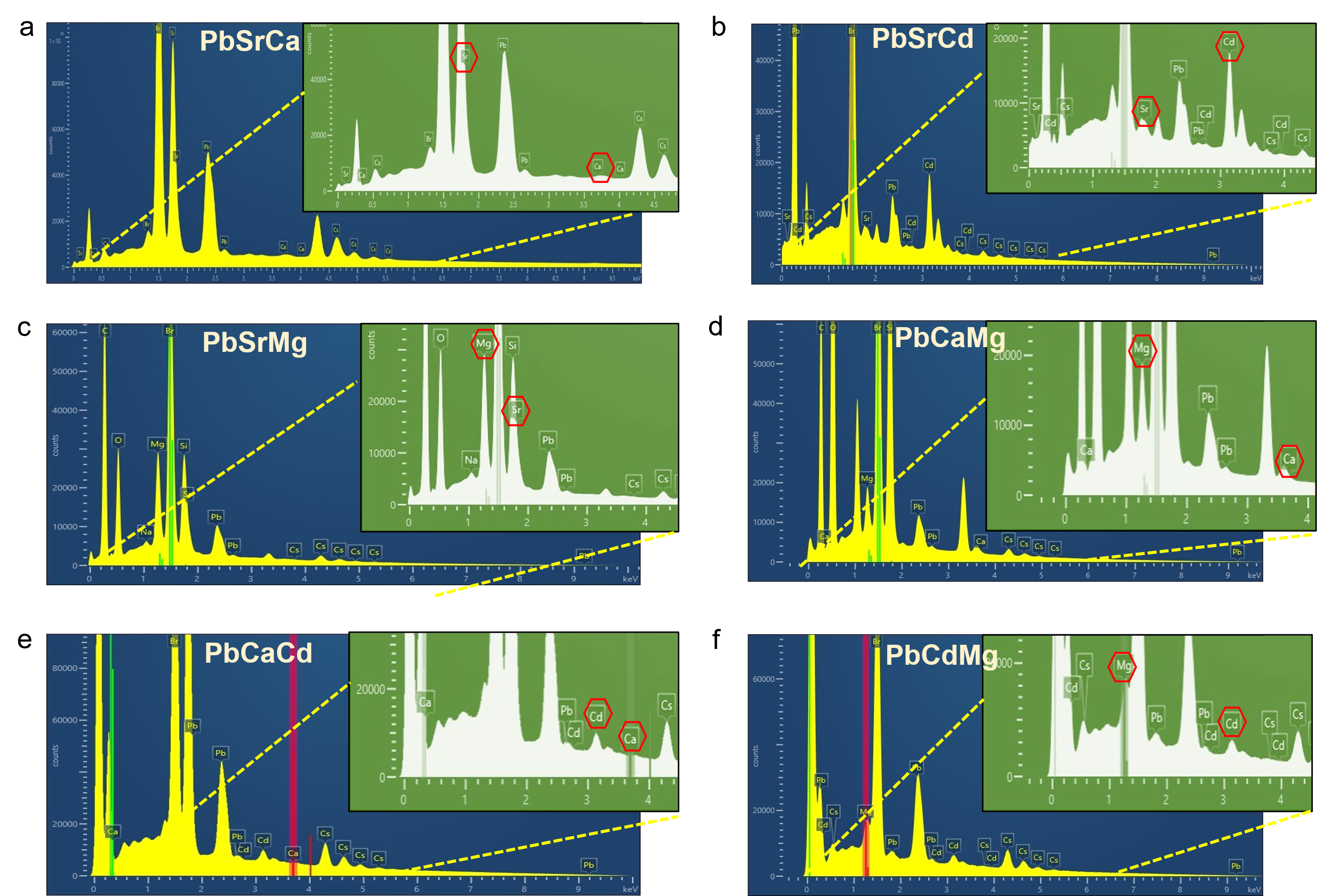


**Figure S7**. The SEM EDS spectrum for trinary alloyed NCs. a) Cs(PbSrCa)Br_3_ NCs. b) Cs(PbSrCd)Br_3_ NCs. c) Cs(PbSrMg)Br_3_ NCs. d) Cs(PbCaMg)Br_3_ NCs. e) Cs(PbCaCd)Br_3_ NCs. f) Cs(PbCdMg)Br_3_ NCs.


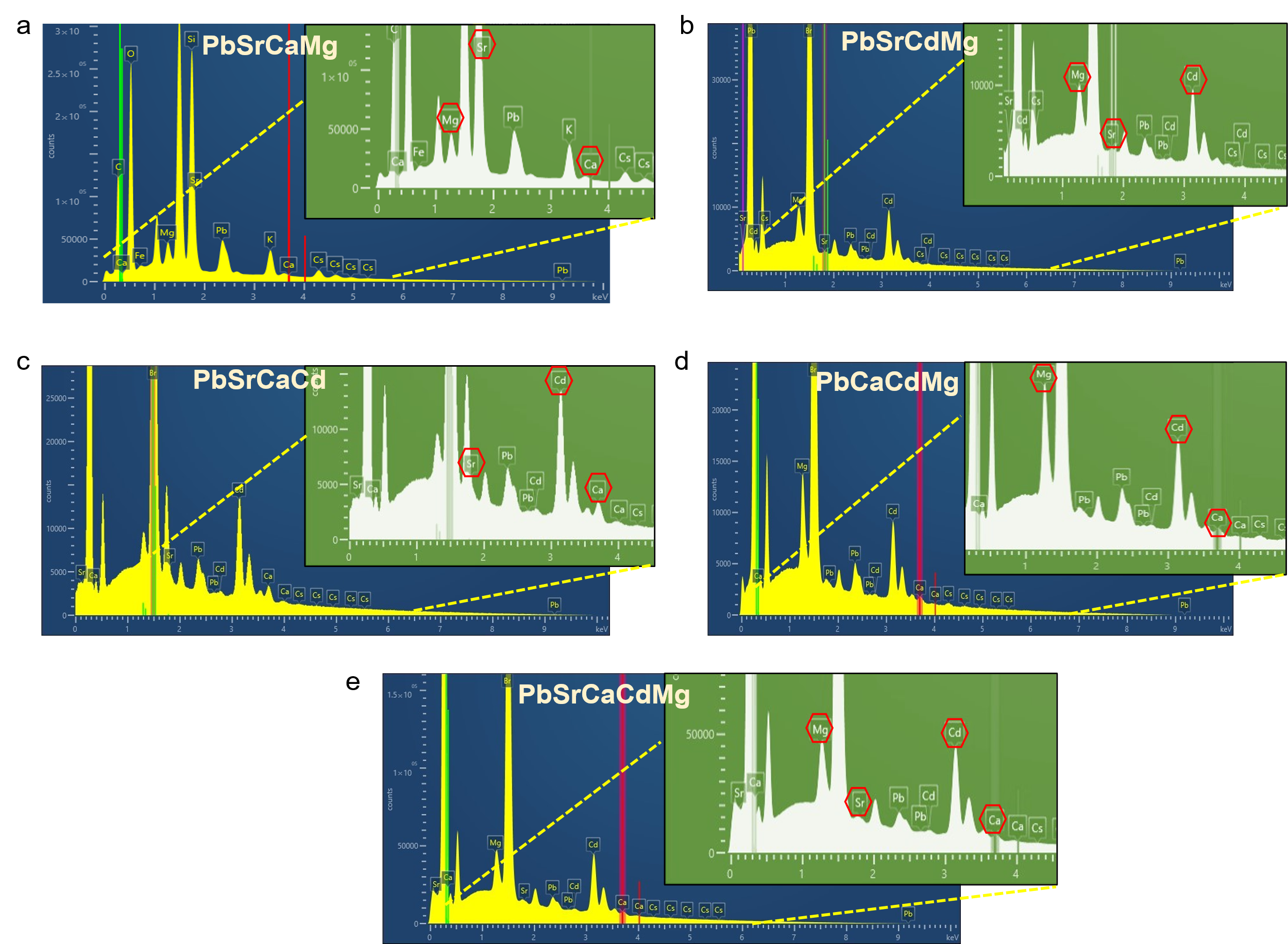


**Figure S8**. The SEM EDS spectrum for quaternary and quinary alloyed NCs. a) Cs(PbSrCaMg)Br_3_ NCs. b) Cs(PbSrCdMg)Br_3_ NCs. c) Cs(PbSrCaCd)Br_3_ NCs. d) Cs(PbCaCdMg)Br_3_ NCs. e) Cs(PbSrCaCdMg)Br_3_ NCs.


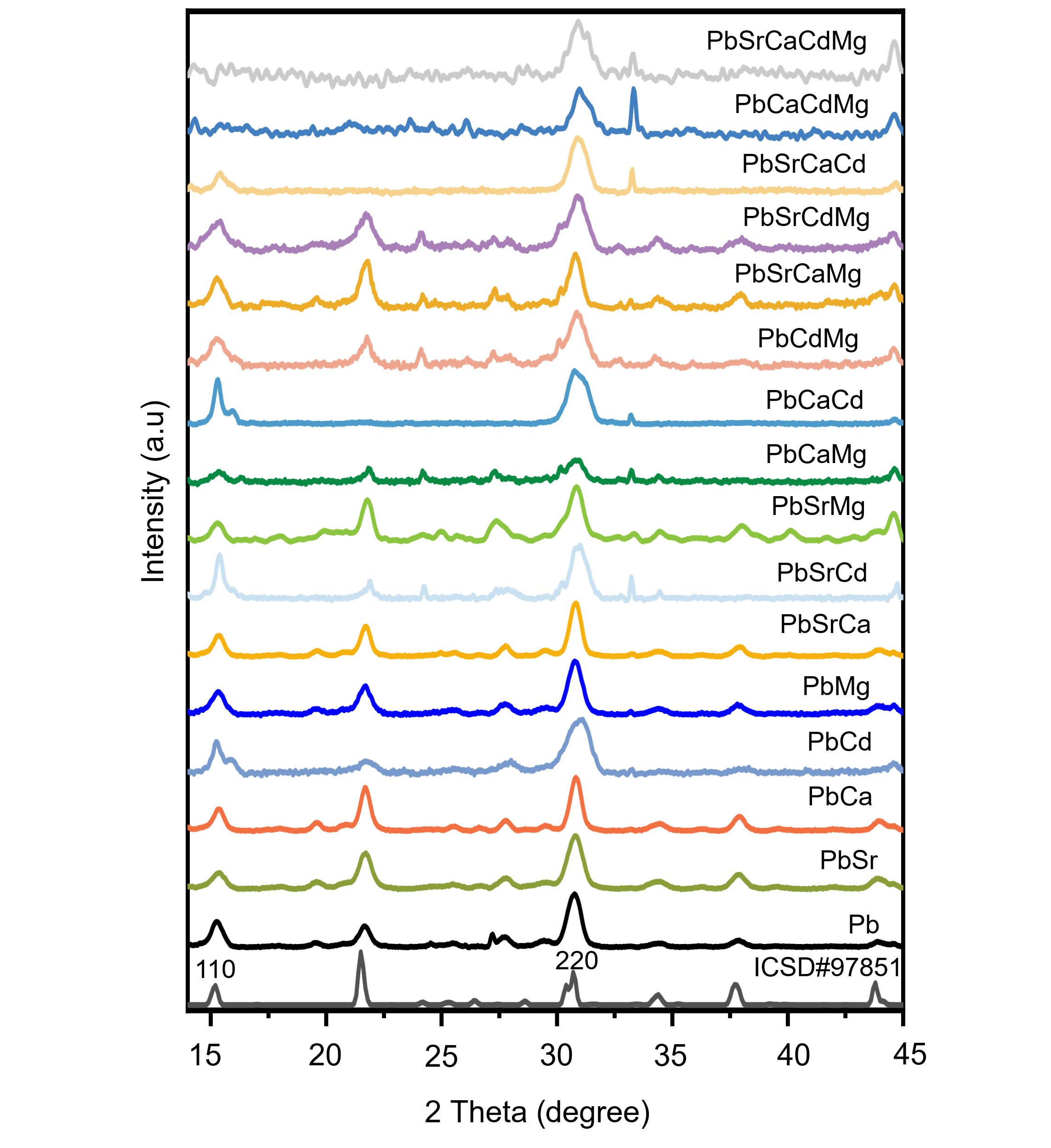


**Figure S9**. XRD patterns of parent, binary, trinary, quaternary and quinary HEP NCs.


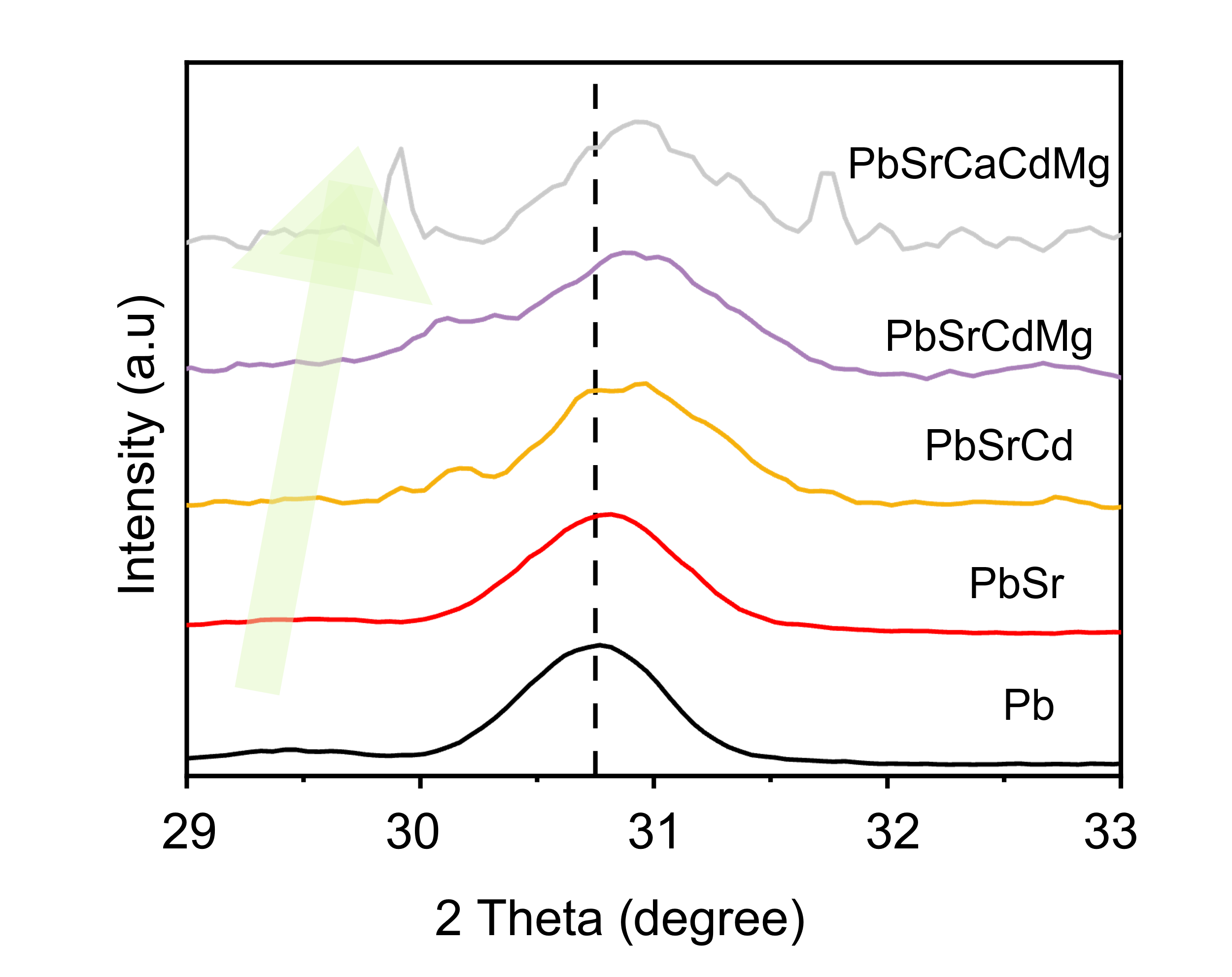


**Figure S10**. Shift in the peak position of (220) plane of HEP NCs.


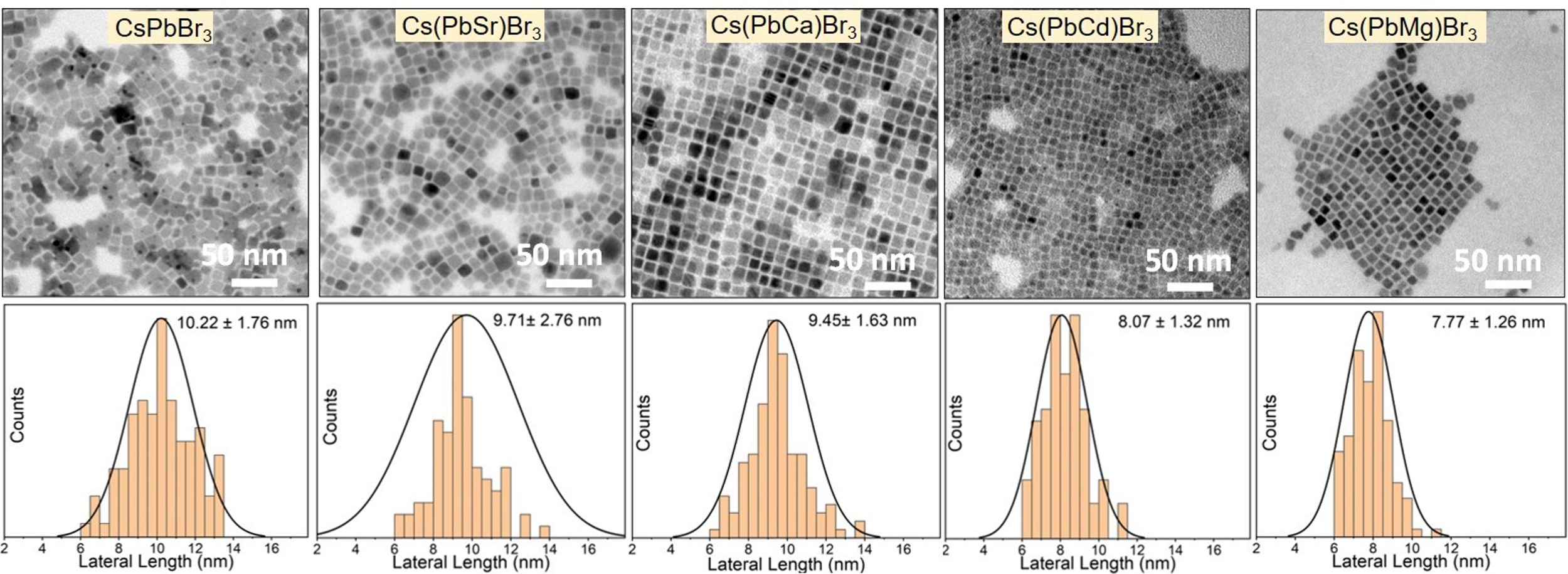


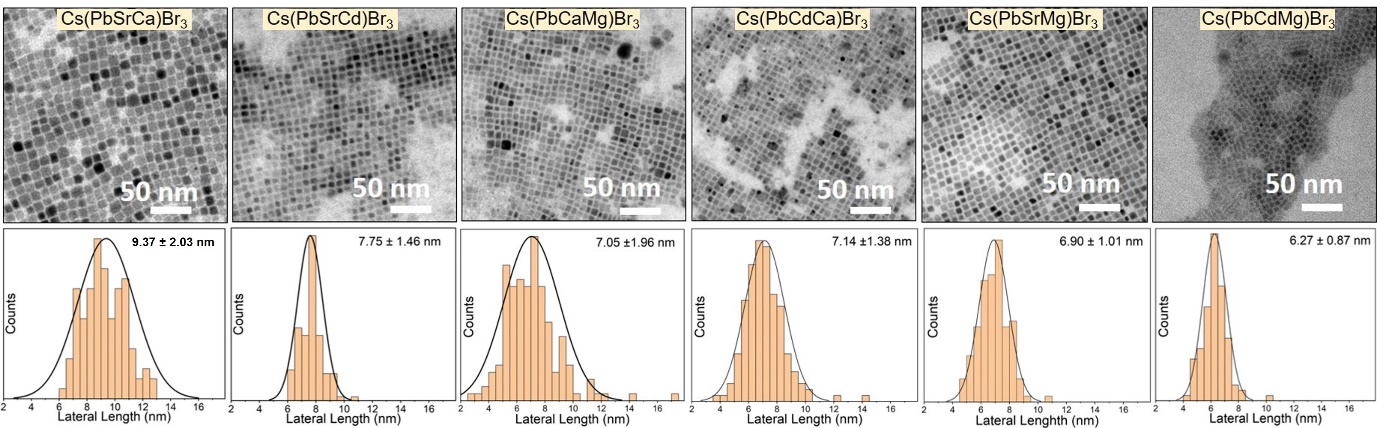


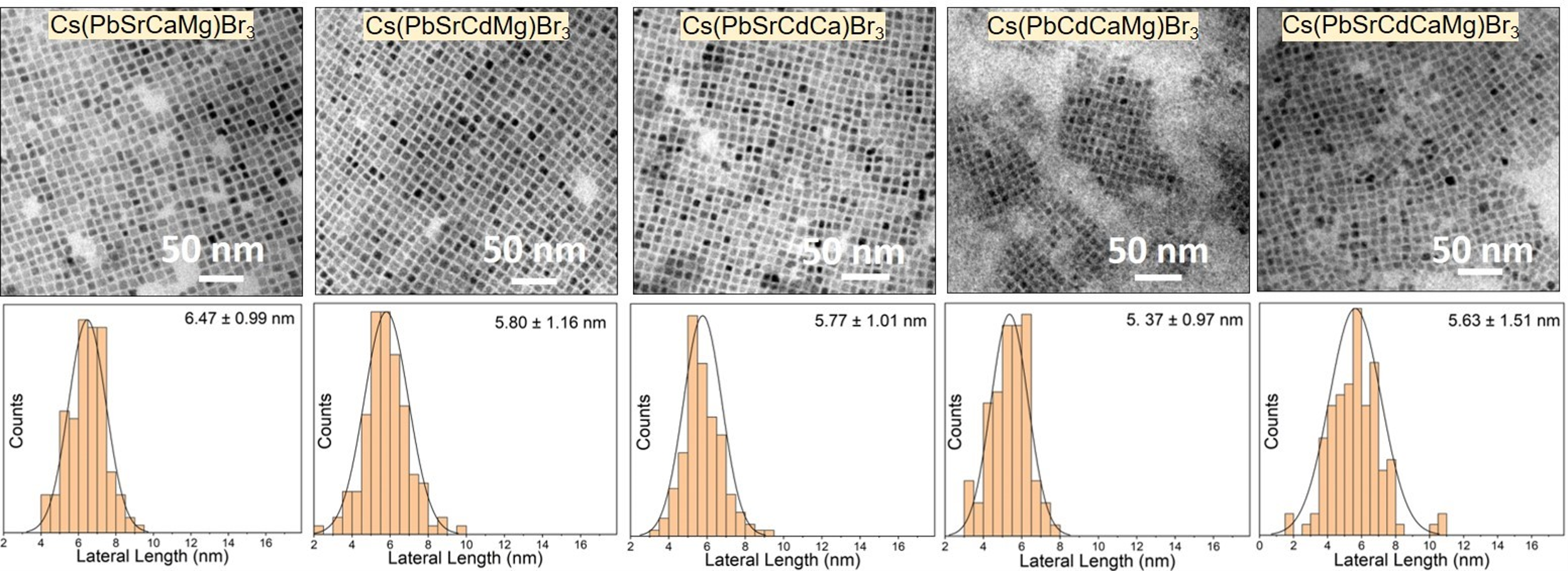


**Figure S11.** TEM images and the corresponding size distribution of HEP NCs.


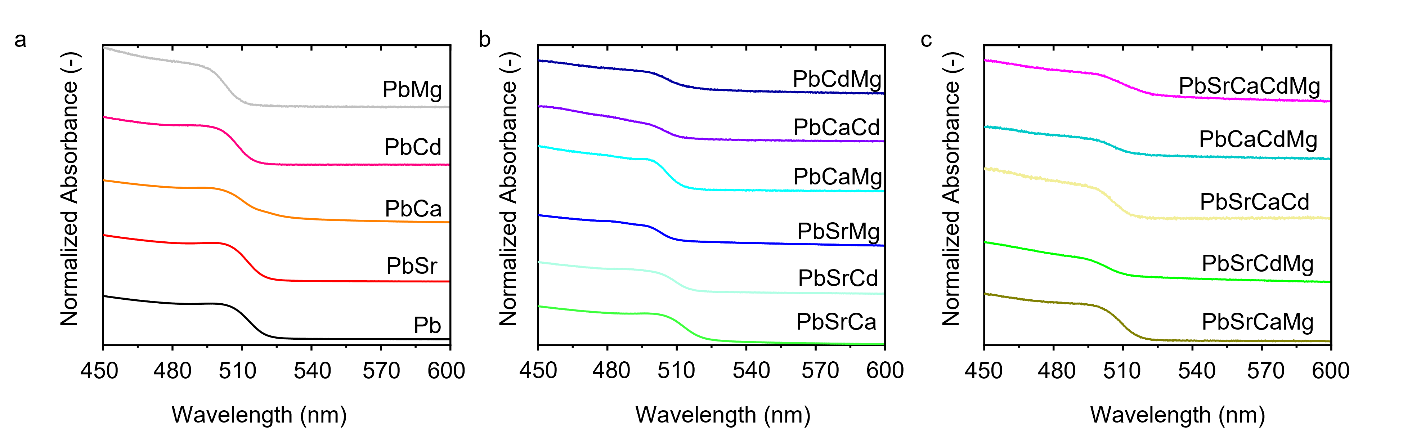


**Figure S12.** Linear absorption spectra of a) pristine and binary alloyed NCs, b) trinary alloyed NCs, c) quaternary and quinary alloyed HEP NCs.


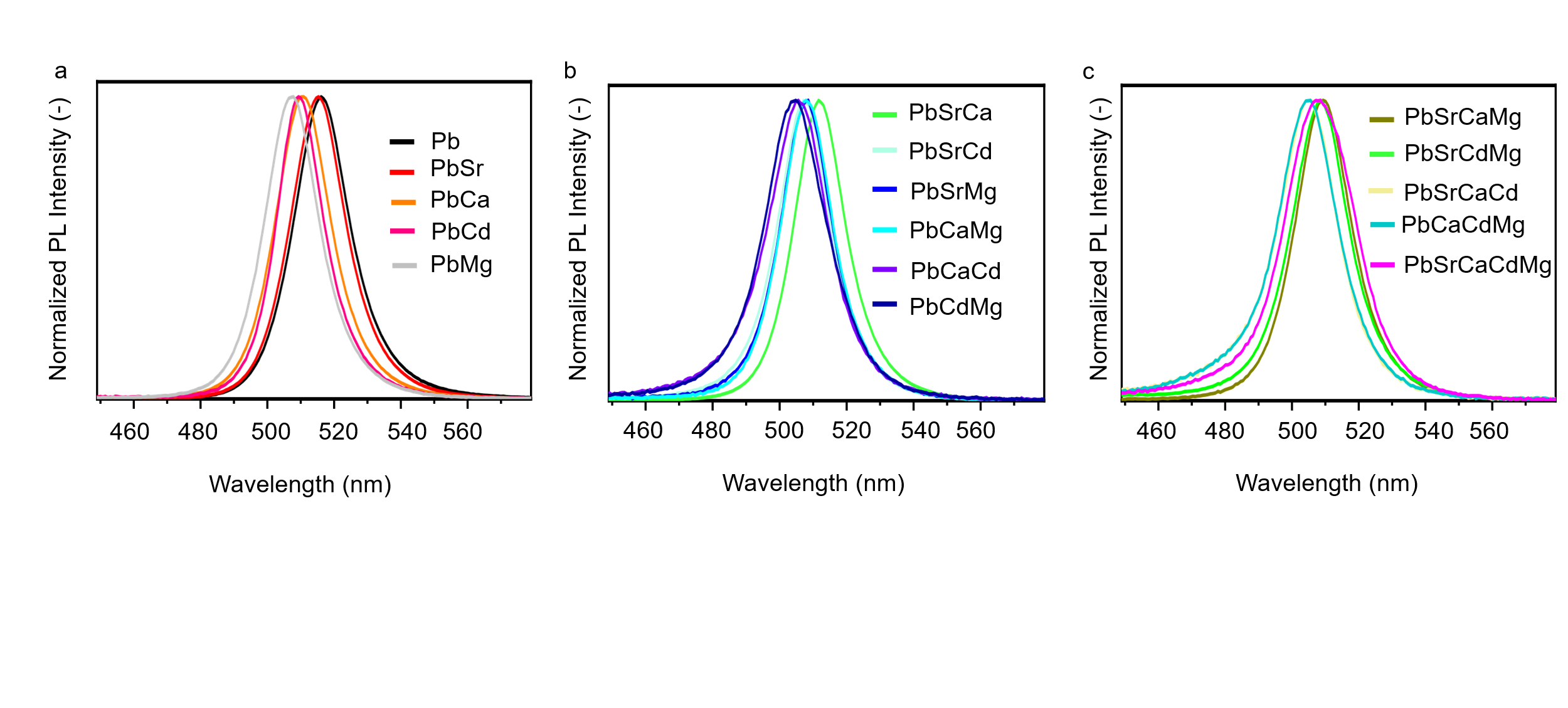


**Figure S13.** Steady state PL spectra of a) parent and binary alloyed NCs, b) trinary alloyed NCs, c) quaternary and quinary alloyed HEP NCs.


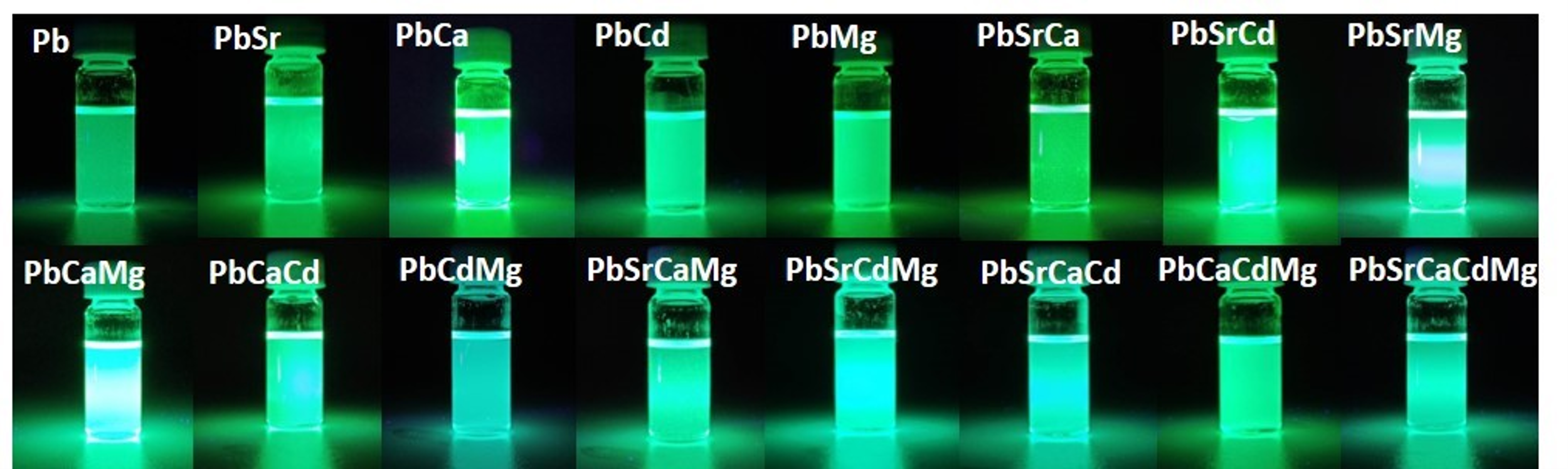


**Figure S14.** The optical images showing the strong fluorescence of all HEP NCs under UV light excitation. The labels denote the number of secondary elements. For example, PbSrCdMg indicates the Cs(PbSrCdMg)Br_3_ NCs.


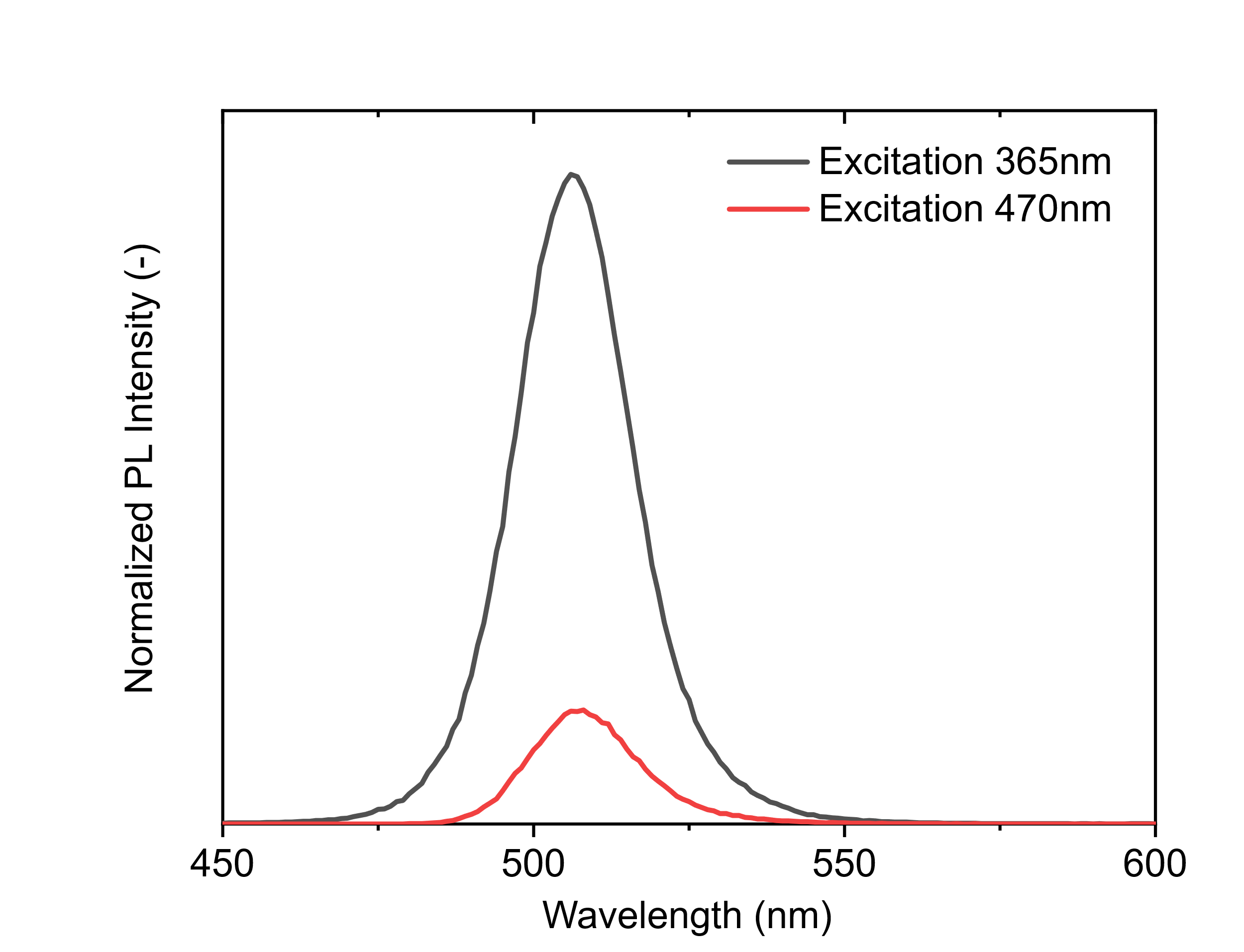


**Figure S15.** PL emission profiles of HEA Cs(PbSrCdMg)Br_3_ NCs excited at 470 nm and 365 nm.


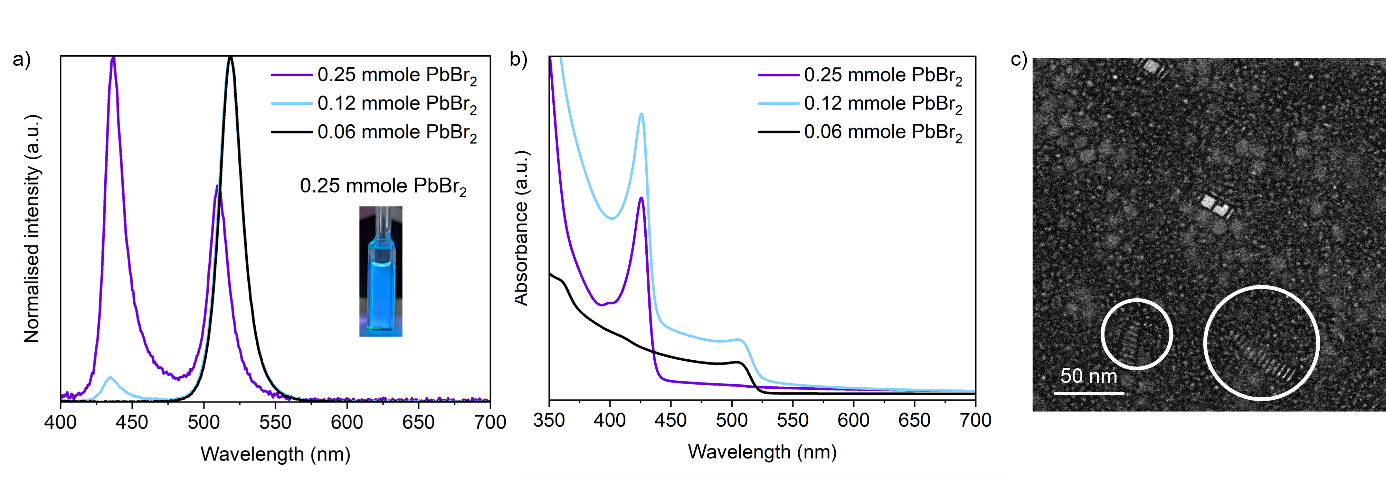


**Figure S16:** a) Photoluminescence spectra, and b) UV-visible absorption profiles of pristine CsPbBr_3_ NCs solution mixed with different amount of PbBr_2_ during the cationic exchange. The η_PL_ after mixed with 0.06mmole, 0.12mmole, 0.25mmole are 25%, 13%, and 5%, respectively. c) Transmission electron micrographs of CsPbBr_3_ after cationic exchange with 0.25mmole PbBr_2_. The circled regions indicate the presence of CspbBr_3_ nanoplatelets.


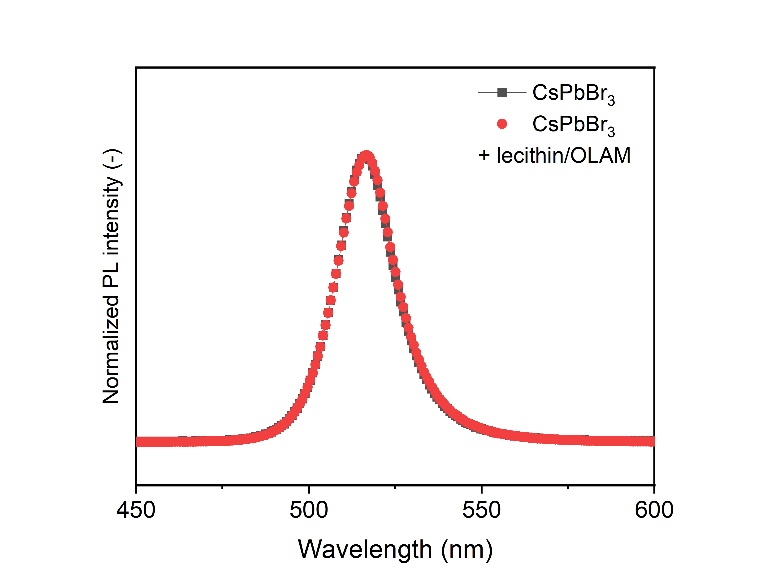


**Figure S17.** PL emission profiles of parent CsPbBr_3_ NCs and CsPbBr_3_ mixed with lecithin and OLAM ligands (CsPbBr_3_ + lecithin/OLAM), showing no hypochromic shift. The *η*_PL_ value of CsPbBr_3_ + lecithin/OLAM is 65%, indicating that the enhanced in HEP NCs results from synergistic interactions between the ligands and alloyed elements.


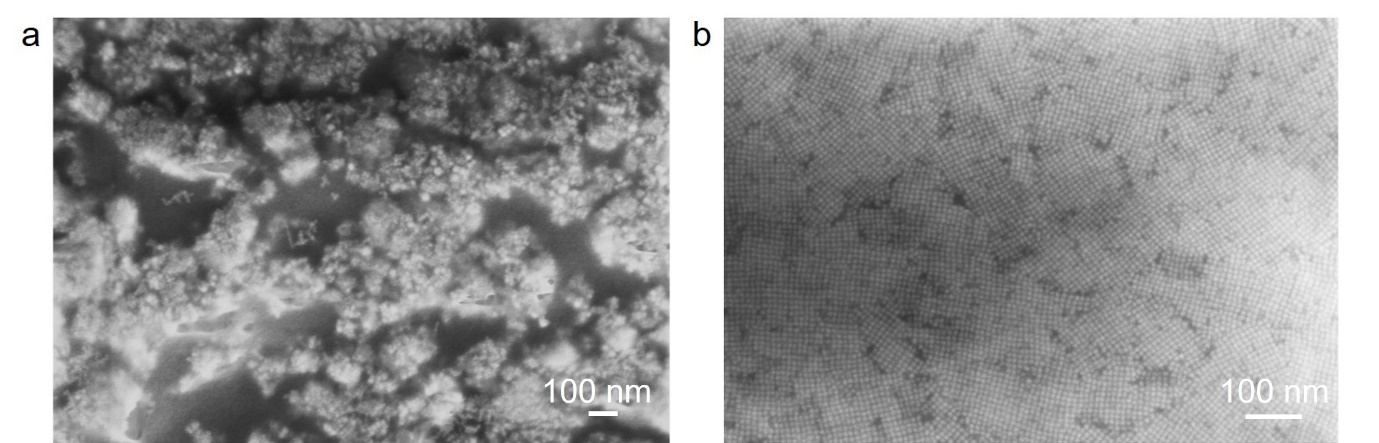


**Figure S18.** a) The SEM image evidence the segregation of parent CsPbBr_3_ NCs after drop casting, showing a high degree of non-uniformity resulting from larger size and low polydispersity. b) The formation of long‑range superlattice from quaternary alloyed Cs(PbSrCdMg)Br_3_ NCs. Under ambient conditions, we found the HEP NCs exhibit a strong tendency to form long-range closed packed superlattice structure in their drop-casted films. We attribute it to the HEA-induced size and shape monodispersity, as well as the reduced size, which facilitate uniform stacking and self-assembly.


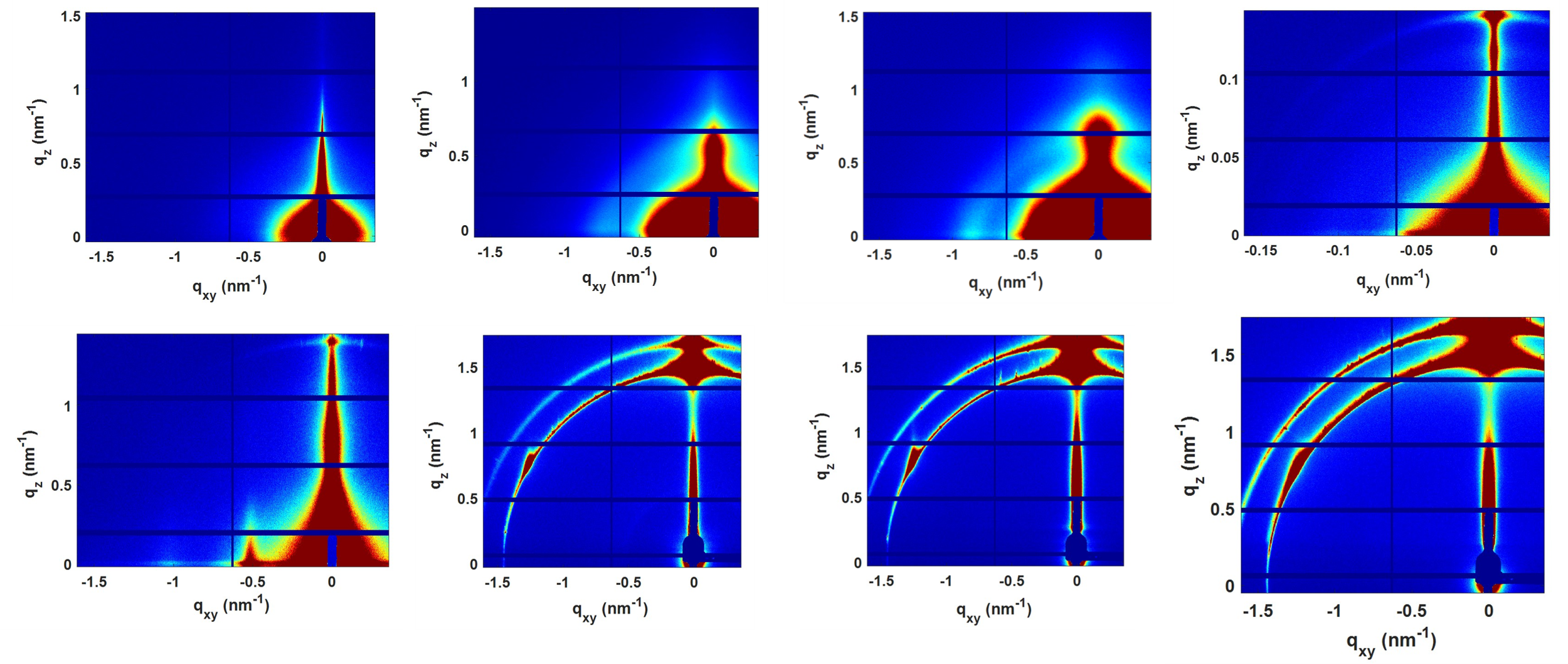


**Figure S19**. GISAX patterns of a) CsPbBr_3_, b) Cs(PbSr)Br_3_, c)Cs(PbCa)Br_3_, d) Cs(PbSrMg)Br_3_, e) Cs(PbCaMg)Br_3_, f) Cs(PbSrCdMg)Br_3_, g)Cs(PbCaCdMg)Br_3_, h) Cs(PbSrCaCdMg)Br_3_ NCs.





**Figure S20**. TRPL spectrum of quantum-confined CsPbBr_3_ NCs.


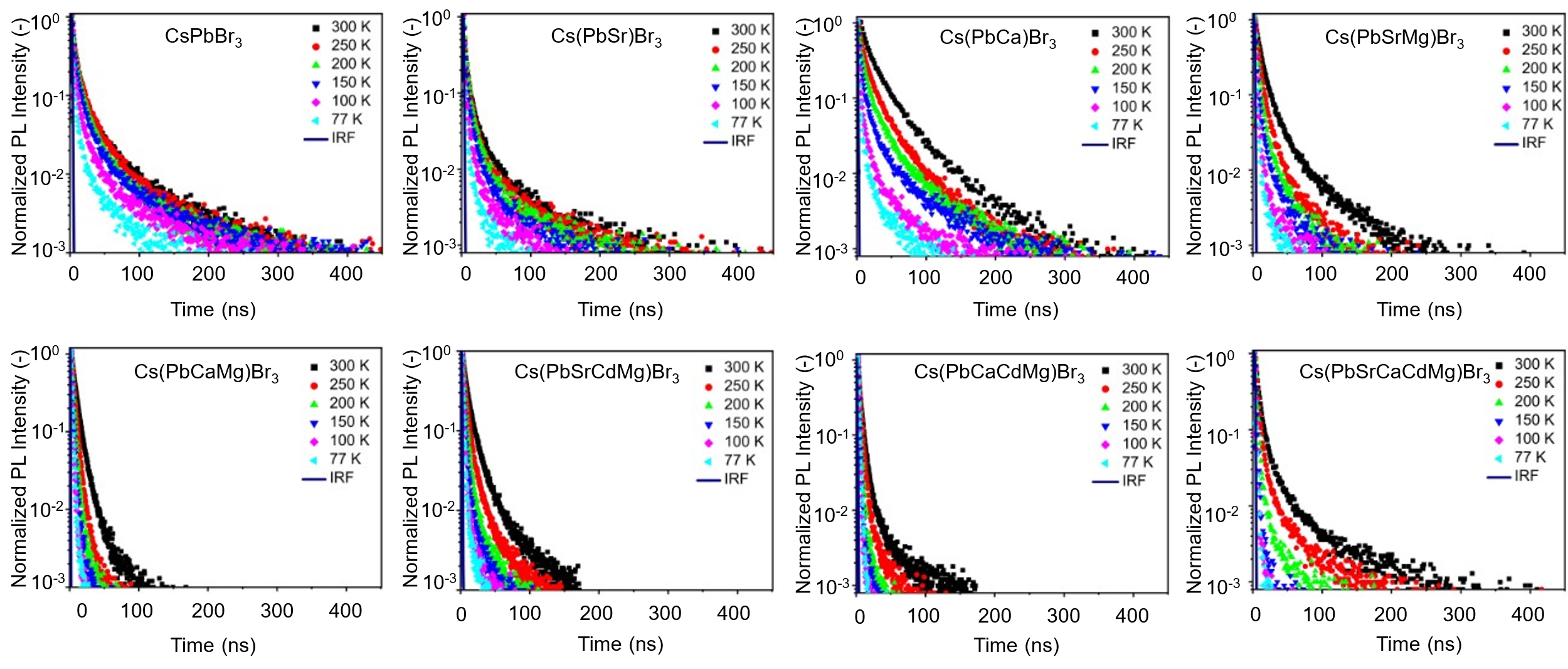


**Figure S21**. Temperature dependent time-resolved PL measurements of single, binary and HEP NCs from 300 K up to 77K. The faster transient PL decay at lower temperatures showcase the enhanced intrinsic band-edge recombination of HEP NCs.


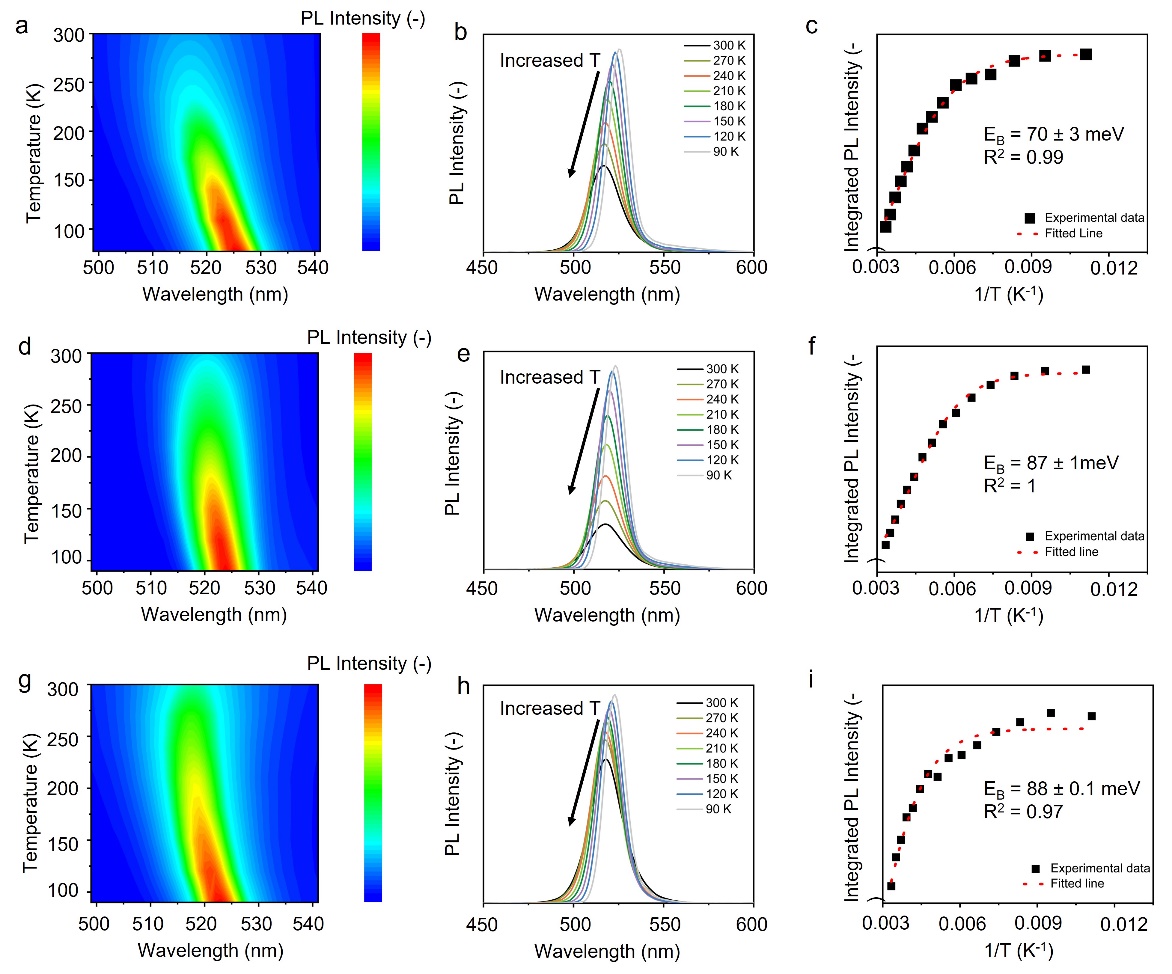


**Figure S22.** Temperature-dependent PL intensity analyses, including contour plot, corresponding steady state PL spectra and extracted exciton binding energies of pristine CsPbBr_3_ (a, b, c), binary alloyed Cs(PbSr)Br_3_ (d, e, f), and Cs(PbCa)Br_3_ (g, h, i). The binding energies are derived from Arrhenius equation (see **Supplementary methods**). The standard deviation is indicated in the respective figure.


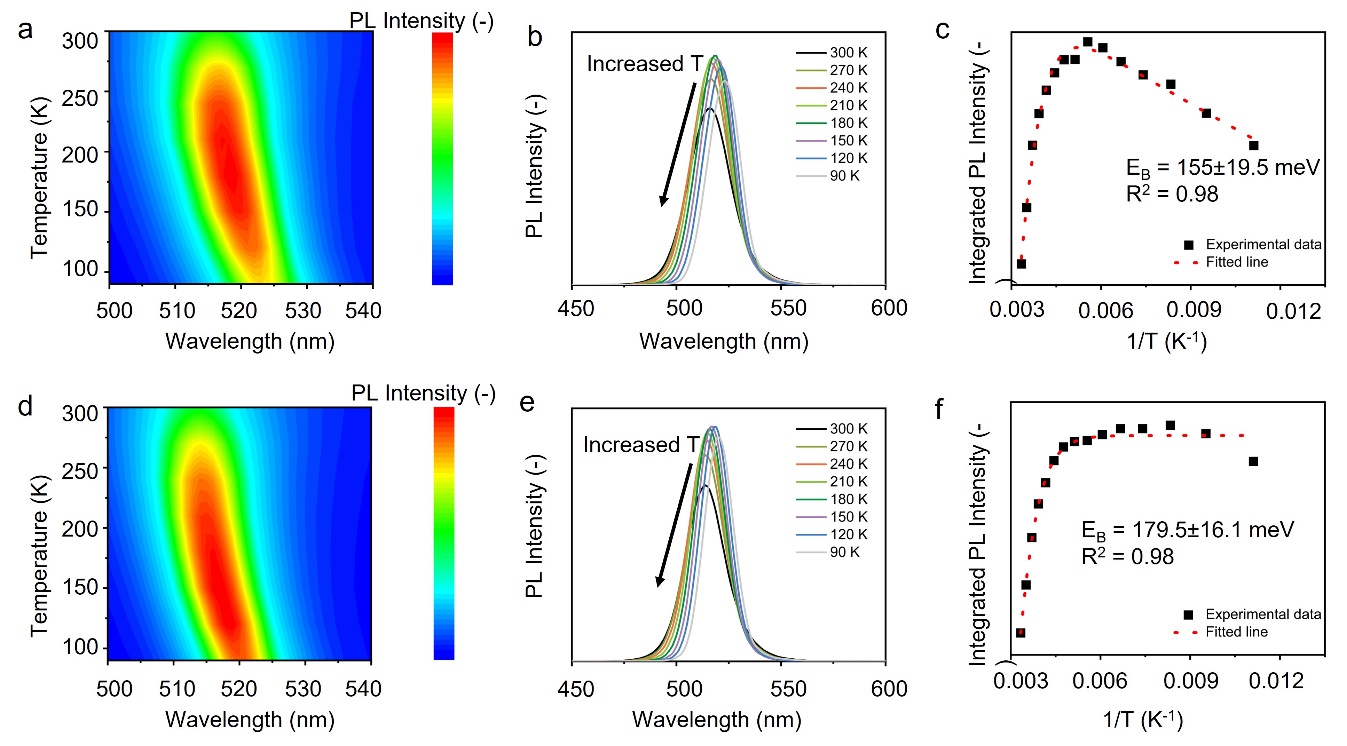


**Figure S23.** Temperature-dependent PL intensity analyses, including contour plot, corresponding PL spectra and extracted exciton binding energies of trinary alloyed Cs(PbSrMg)Br_3_ (a, b, c) and Cs(PbCaMg)Br_3_ (d, e, f) NCs. The binding energies are derived from Arrhenius equation (see **Supplementary methods**). The standard deviation is indicated in respective figures.


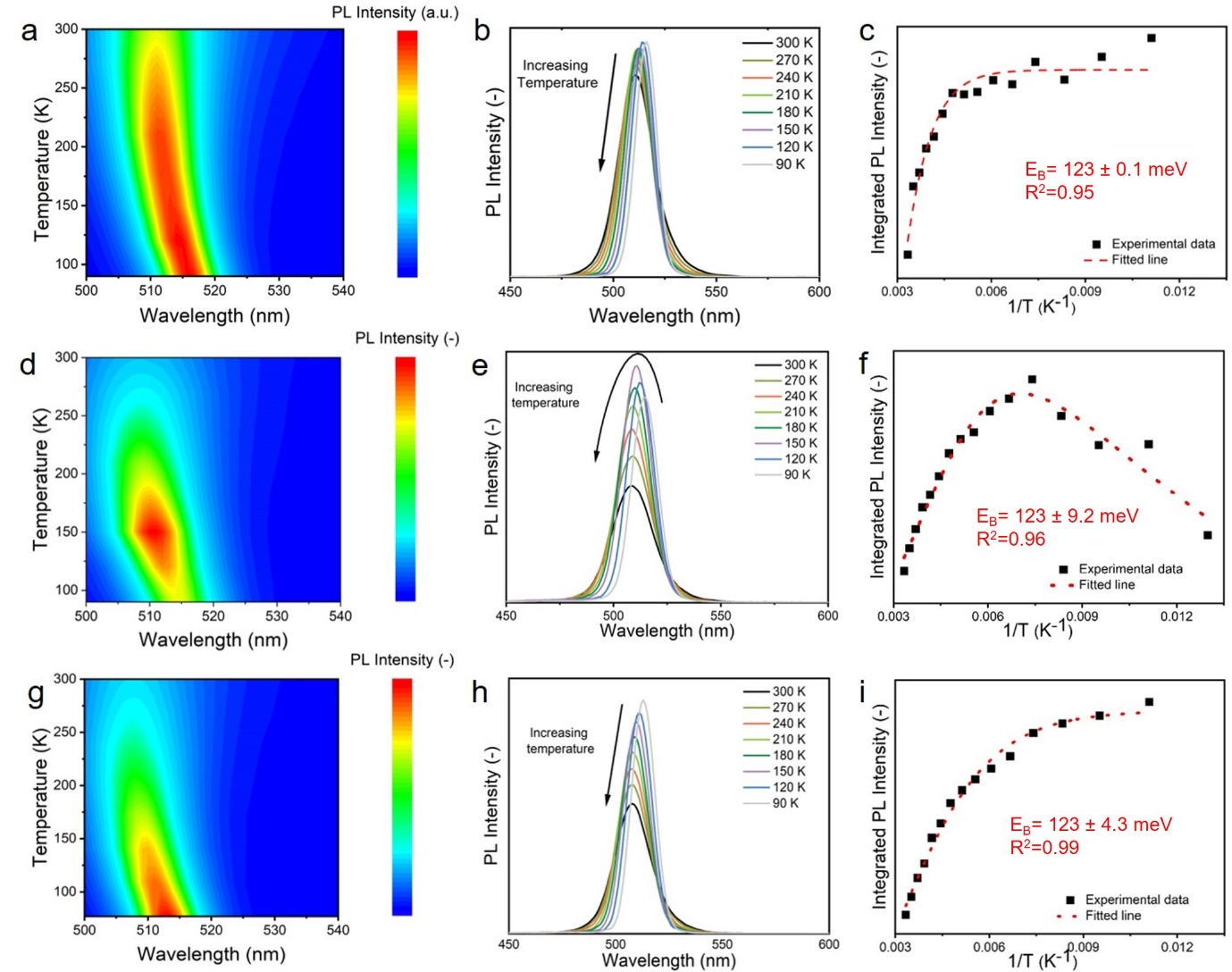


**Figure** **S24.** Temperature-dependent PL intensity analyses, including contour plots, corresponding PL spectra and extracted exciton binding energies of quaternary and quinary alloyed Cs(PbSrCdMg)Br_3_ (a, b, c), Cs(PbCaCdMg)Br_3_ (d, e, f) and Cs(PbSrCaCdMg)Br_3_ NCs. The binding energies are derived from Arrhenius equation (see **Supplementary methods**). The standard deviation is indicated in respective figures.


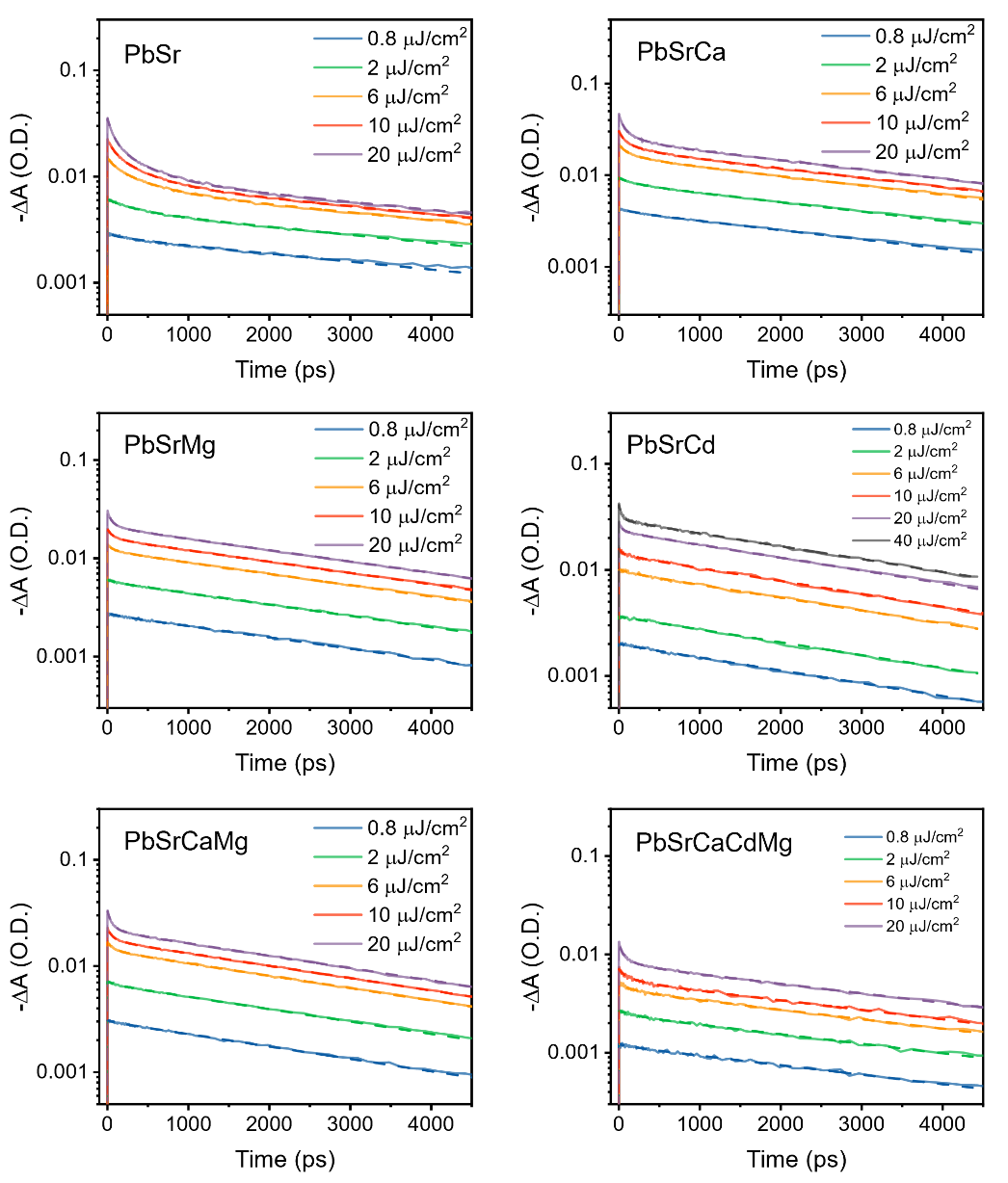


**Figure S25**. Kinetics probed at ground state bleaching (GSB) (dash lines are global fitting curves of three exponential functions deconvolved with instrument response function). The time constants are treated as the shared parameters while the corresponding amplitudes are fluence-dependent).


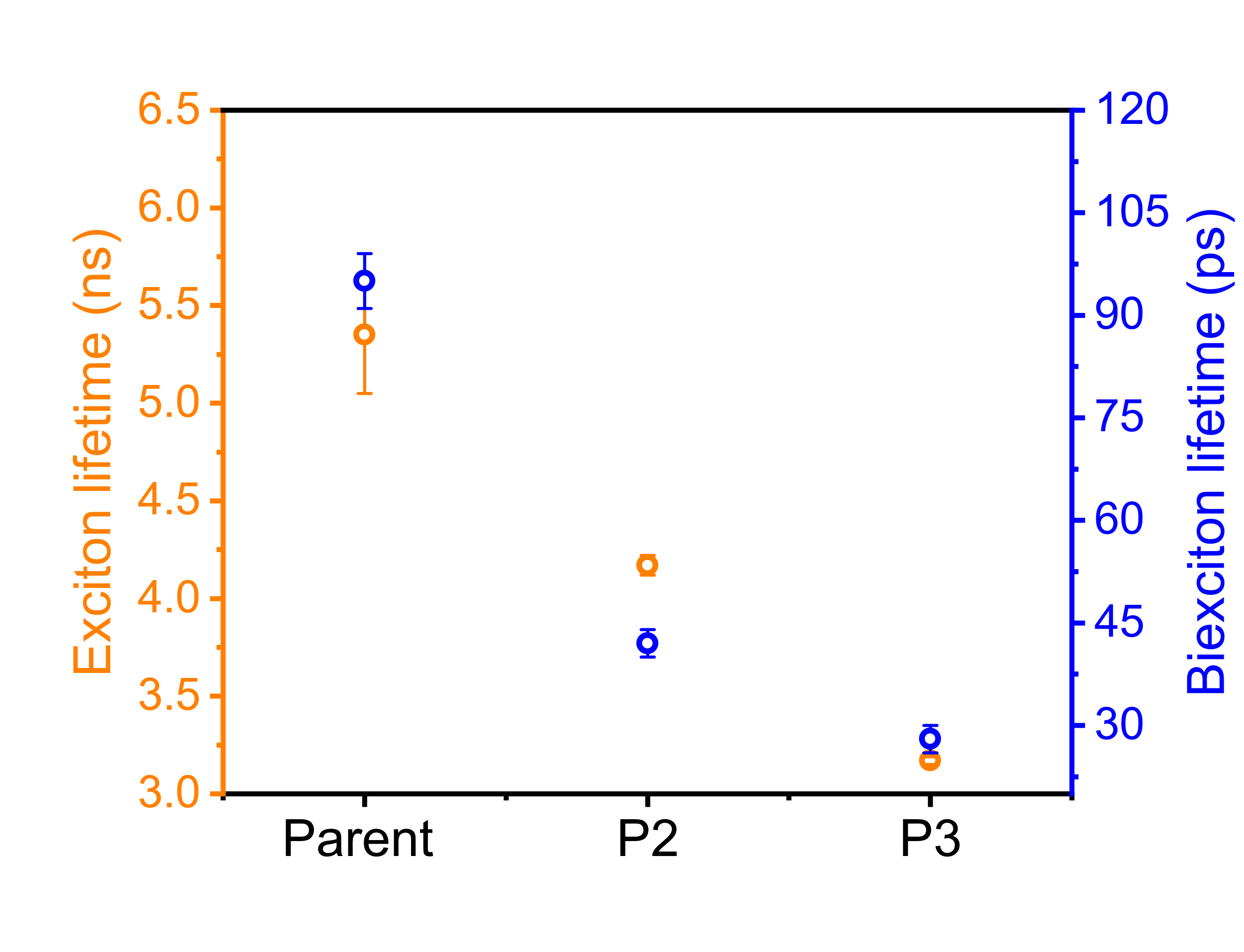


**Figure S26**. Exciton and biexciton lifetimes of CsPbBr_3_ NCs with different sizes (Parent—10 nm, P2—8 nm and P3—6 nm).

**
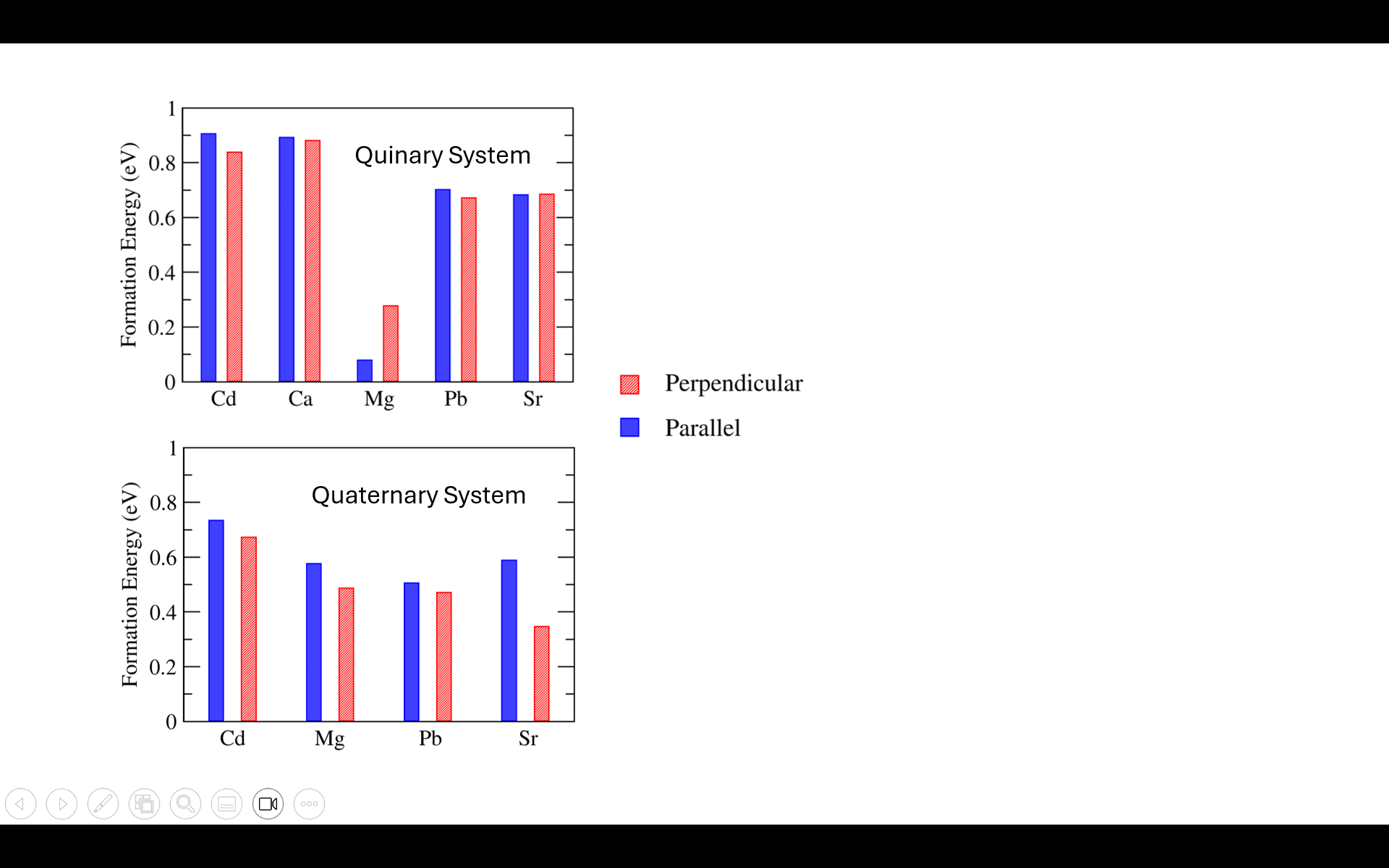
**

**Figure S27.** Formation energy of an XBr_2_ vacancy within the quinary and quaternary HEA systems where X = Cd, Ca, Mg, Pb, Sr. The X-Br bonds of the vacated atoms can be perpendicular (red bars) or parallel (blue bars) to each other.


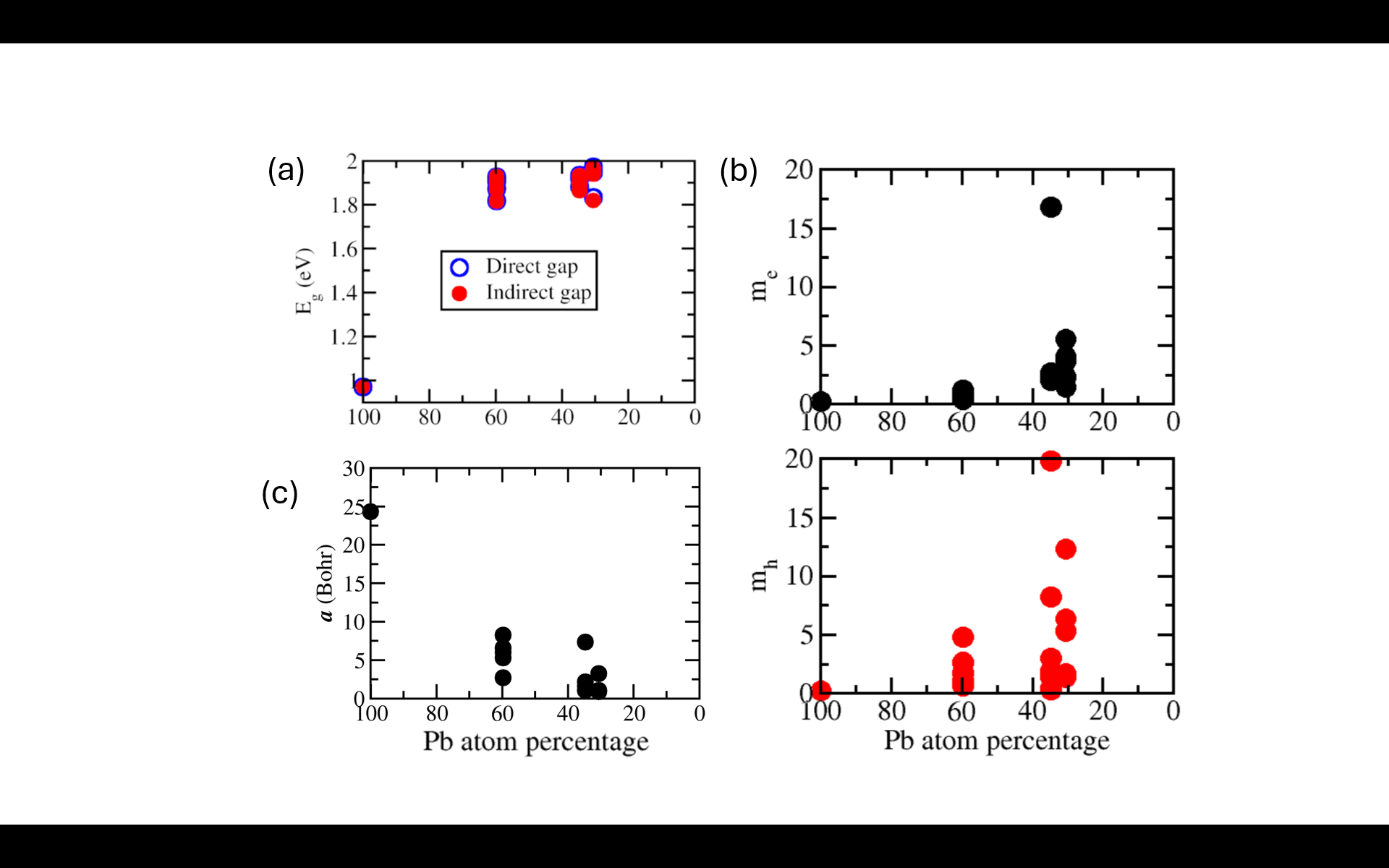


**Figure S28**. a) Direct and indirect band gaps, b) effective masses, as well as c) Bohr radii obtained from our calculations on trinary alloyed and HEP NCs.

| a) |  | b) |  |
| --- | --- | --- | --- |
| c) |  |  |  |

**Figure S29.** XPS analysis of CsPbBr_3_ NCs. a) Pb 4f region, b) Br 3p region, and c) Cs 3d_5/2_ region.

| a) |  | b) |  |
| --- | --- | --- | --- |
| c) |  |  |  |

**Figure S30.** XPS analysis of Cs(PbSr)Br_3_ NCs. a) Pb 4f region, b) Br 3p region, and c) Cs 3d_5/2_ region.

| a) |  | b) |  |
| --- | --- | --- | --- |
| c) |  |  |  |

**Figure S31.** XPS analysis of Cs(PbCd)Br_3_ NCs. a) Pb 4f region, b) Br 3p region, and c) Cs 3d_5/2_ region.

| a) |  | b) |  |
| --- | --- | --- | --- |
| c) |  |  |  |

**Figure S32.** XPS analysis of Cs(PbCa)Br_3_ NCs. a) Pb 4f region, b) Br 3p region, and c) Cs 3d_5/2_ region.

| a) |  | b) |  |
| --- | --- | --- | --- |
| c) |  |  |  |

**Figure S33.** XPS analysis of Cs(PbMg)Br_3_ NCs. a) Pb 4f region, b) Br 3p region, and c) Cs 3d_5/2_ region.

| a) |  | b) |  |
| --- | --- | --- | --- |
| c) |  |  |  |

**Figure S34.** XPS analysis of Cs(PbSrCa)Br_3_ NCs. a) Pb 4f region, b) Br 3p region, and c) Cs 3d_5/2_ region.

| a) |  | b) |  |
| --- | --- | --- | --- |
| c) |  |  |  |

**Figure S35.** XPS analysis of Cs(PbSrCd)Br_3_ NCs. a) Pb 4f region, b) Br 3p region, and c) Cs 3d_5/2_ region.

| a) |  | b) |  |
| --- | --- | --- | --- |
| c) |  |  |  |

**Figure S36.** XPS analysis of Cs(PbSrMg)Br_3_ NCs. a) Pb 4f region, b) Br 3p region, and c) Cs 3d_5/2_ region.

| a) |  | b) |  |
| --- | --- | --- | --- |
| c) |  |  |  |

**Figure S37.** XPS analysis of Cs(PbCaMg)Br_3_ NCs. a) Pb 4f region, b) Br 3p region, and c) Cs 3d_5/2_ region.

| a) |  | b) |  |
| --- | --- | --- | --- |
| c) |  |  |  |

**Figure S38.** XPS analysis of Cs(PbCaCd)Br_3_ NCs. a) Pb 4f region, b) Br 3p region, and c) Cs 3d_5/2_ region.

| a) |  | b) |  |
| --- | --- | --- | --- |
| c) |  |  |  |

**Figure S39.** XPS analysis of Cs(PbSrCaMg)Br_3_ NCs. a) Pb 4f region, b) Br 3p region, and c) Cs 3d_5/2_ region.

| a) |  | b) |  |
| --- | --- | --- | --- |
| c) |  |  |  |

**Figure S40.** XPS analysis of Cs(PbSrCdMg)Br_3_ NCs. a) Pb 4f region, b) Br 3p region, and c) Cs 3d_5/2_ region.

| a) |  | b) |  |
| --- | --- | --- | --- |
| c) |  |  |  |

**Figure S41.** XPS analysis of Cs(PbSrCdCa)Br_3_ NCs. a) Pb 4f region, b) Br 3p region, and c) Cs 3d_5/2_ region.

| a) |  | b) |  |
| --- | --- | --- | --- |
| c) |  |  |  |

**Figure S42.** XPS analysis of Cs(PbCdCaMg)Br_3_ NCs. a) Pb 4f region, b) Br 3p region, and c) Cs 3d_5/2_ region.

| a) |  | b) |  |
| --- | --- | --- | --- |
| c) |  |  |  |

**Figure S43.** XPS analysis of Cs(PbSrCdCaMg)Br_3_ NCs. a) Pb 4f region, b) Br 3p region, and c) Cs 3d_5/2_ region.

| a) | 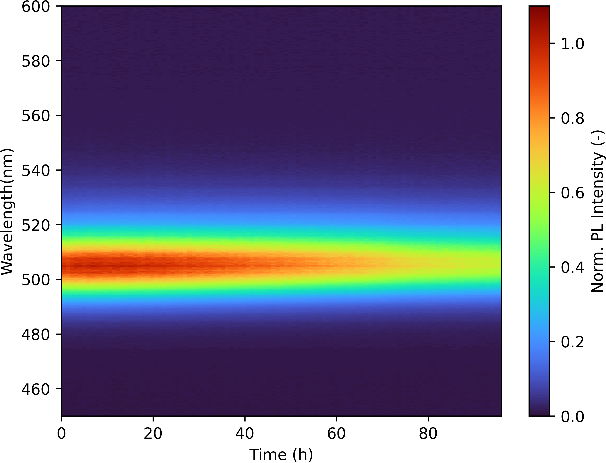 | b) | 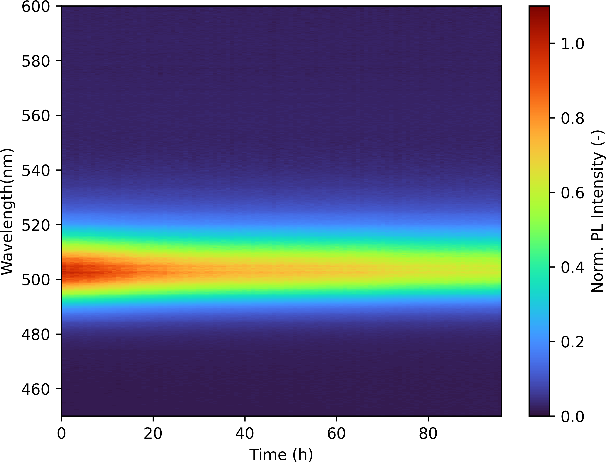 |
| --- | --- | --- | --- |
| c) | 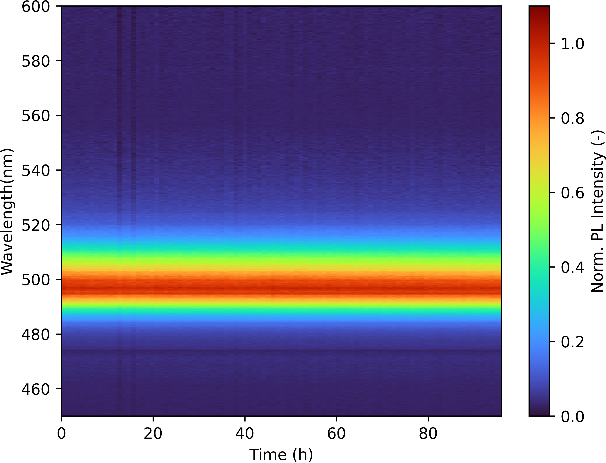 | d) | 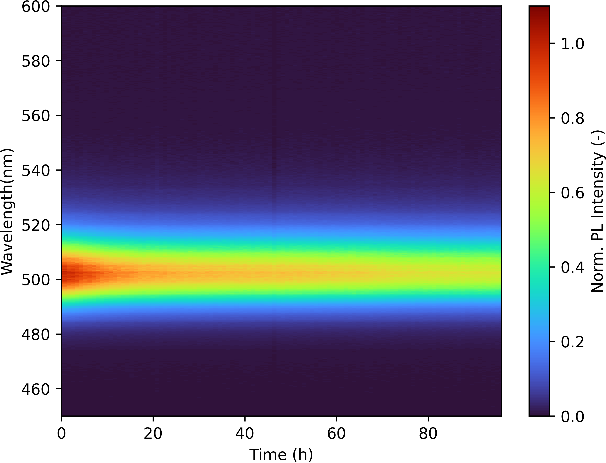 |
| e) | 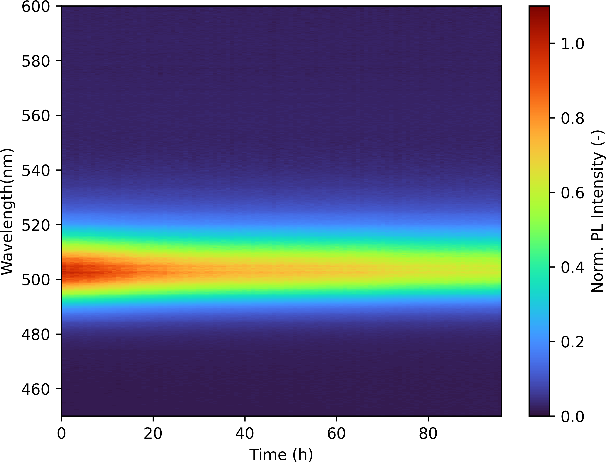 | f) | 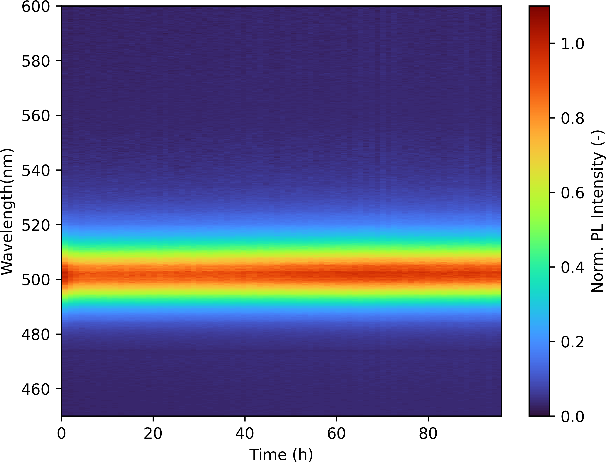 |
| g) | 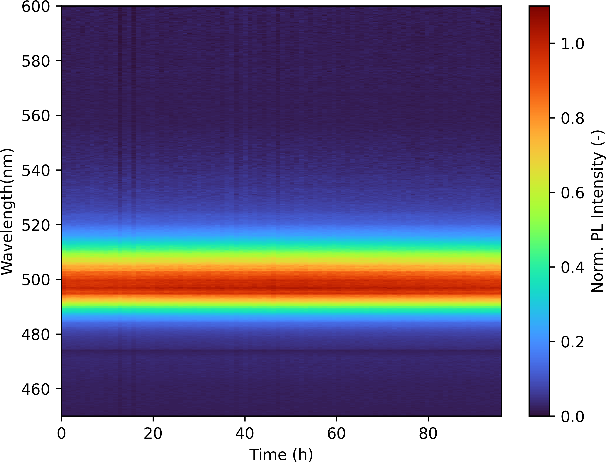 | h) | 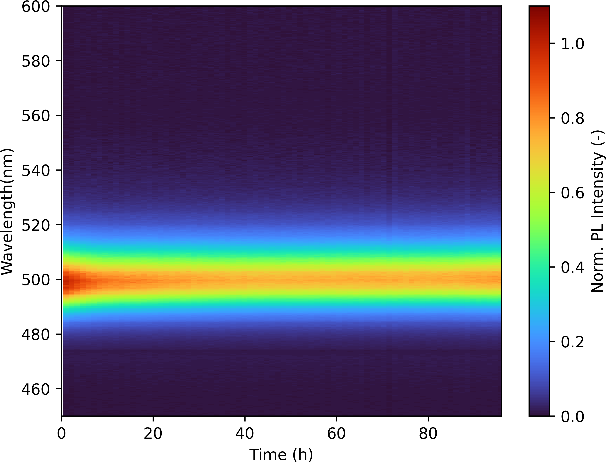 |
| i) | 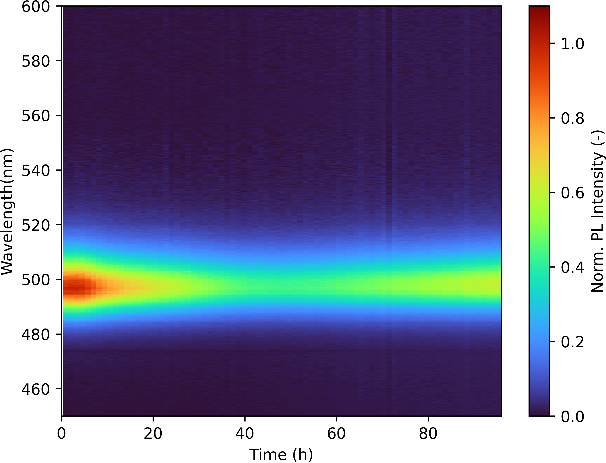 | j) | 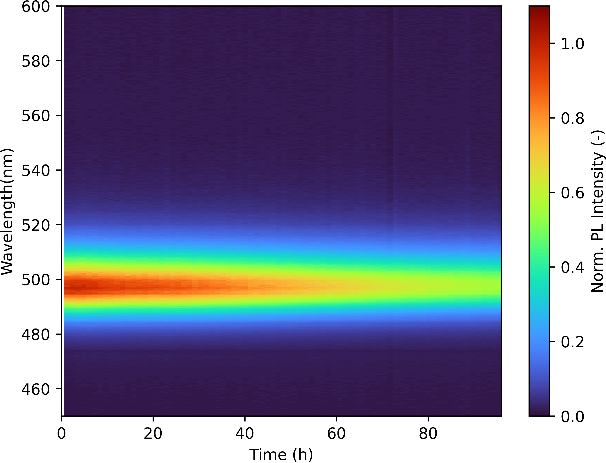 |
| k) | 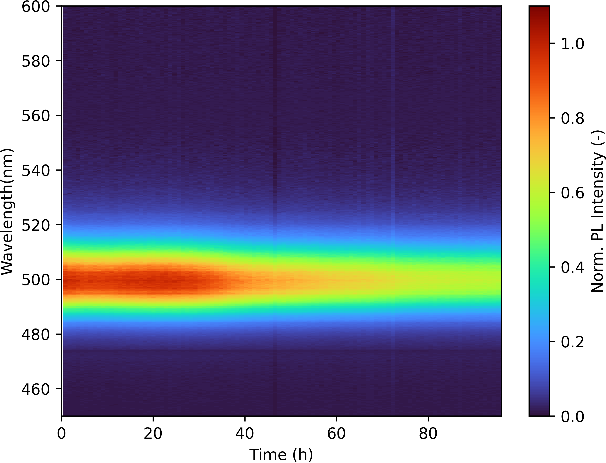 | l) | 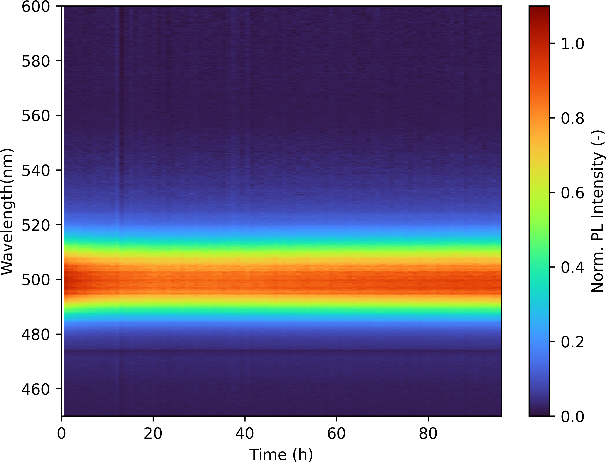 |
| m) | 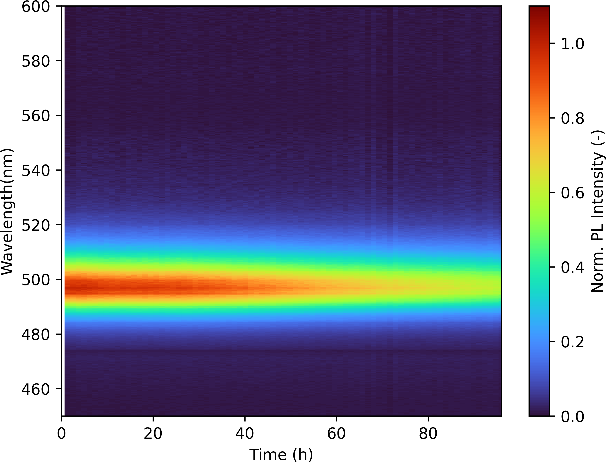 | n) | 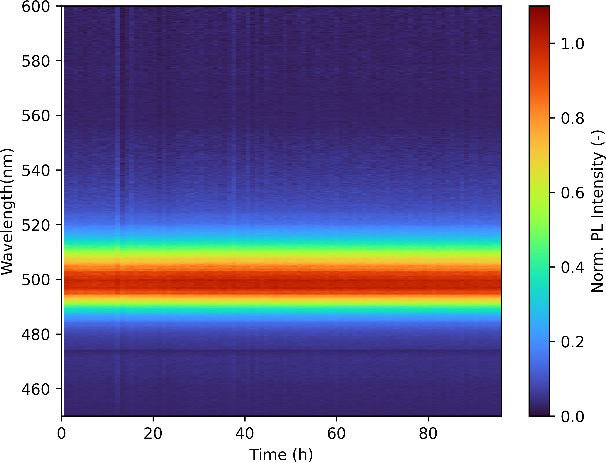 |
| o) | 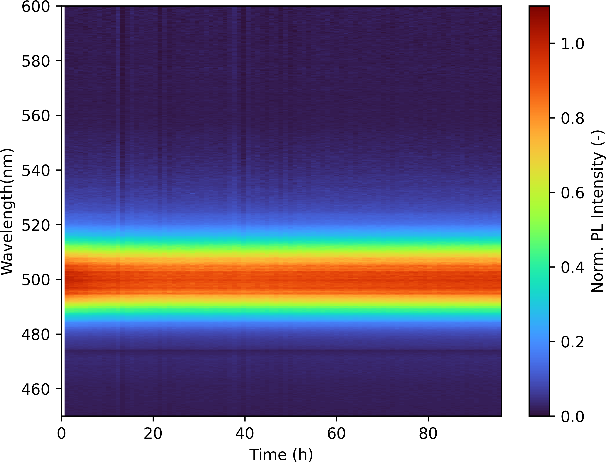 |  |  |

**Figure S44.** *In-situ* PL aging results under ambient conditions (25 °C, 60 – 70% rel. humidity) for Cs*X*Br_3_, where *X*= a)Pb b)PbSr c)PbCd d)PbCa e)PbMg f)PbSrCa g)PbSrCd h)PbSrMg i)PbCaMg j)PbCaCd k)PbSrCaMg l)PbSrCdMg m)PbSrCdCa n)PbCdCaMg o)PbSrCdCaMg.

**Table S1.** The atomic ratio (%) from high-resolution STEM-EDS elemental analysis for parent and selective HEP NCs. The corresponding spectrum is presented in **Figure S1-S5.**

| B-site metals | Cs | Pb | Br | Sr | Ca | Cd | Mg |
| --- | --- | --- | --- | --- | --- | --- | --- |
| Pb | 19.80 | 17.90 | 62.30 | - | - | - | - |
| PbSrCdMg | 14.05 | 14.47 | 64.62 | N.D. | - | 5.09 | 1.77 |
| PbCaCdMg | 13.28 | 15.15 | 61.78 | - | 1.67 | 6.17 | 1.95 |
| PbSrCaCdMg | 14.73 | 12.46 | 61.58 | N.D. | 0.97 | 8.08 | 2.19 |

**Table S2.** The atomic ratio (%) from SEM-EDS elemental analysis for parent, binary, trinary, and HEP NCs thin film studied in this work. The corresponding spectrum is presented in **Figure S6 to S8**.

| B-site metals | Cs | Pb | Br | Sr | Ca | Cd | Mg |
| --- | --- | --- | --- | --- | --- | --- | --- |
| Pb | 19.79 | 19.44 | 60.77 | - | - | - | - |
| PbSr | 19.60 | 19.60 | 56.80 | 4.00 | - | - | - |
| PbCa | 21.83 | 21.84 | 55.70 | - | 0.63 | - | - |
| PbCd | 17.94 | 11.96 | 62.53 | - | - | 7.57 | - |
| PbMg | 18.75 | 18.85 | 58.75 | - | - | - | 5.78 |
| PbSrCa | 16.49 | 17.53 | 61.34 | 4.12 | 5.16 | - | - |
| PbSrCd | 20.39 | 12.53 | 60.82 | 1.02 | - | 5.23 | - |
| PbSrMg | 15.57 | 20.02 | 51.30 | 2.40 | - | - | 17.11 |
| PbCaCd | 18.14 | 16.98 | 59.07 | - | 1.40 | 4.42 | - |
| PbCaMg | 17.91 | 15.96 | 55.33 | - | 4.36 | - | 9.70 |
| PbCdMg | 14.88 | 10.36 | 51.07 | - | - | 10.83 | 17.16 |
| PbSrCaMg | 16.67 | 15.63 | 54.17 | 2.08 | 1.04 | - | 8.21 |
| PbSrCdMg | 17.05 | 10.67 | 52.82 | 1.57 | - | 10.56 | 10.33 |
| PbSrCaCd | 18.21 | 12.29 | 55.20 | 0.89 | 4.35 | 9.05 | - |
| PbCaCdMg | 17.30 | 12.41 | 54.95 | - | 1.67 | 7.20 | 9.18 |
| PbSrCaCdMg | 16.50 | 9.10 | 57.45 | 1.02 | 3.30 | 8.19 | 6.03 |

**Table S3.** The atomic ratio (%) from SEM-EDS elemental analysis of selected crude HEP NCs solution. The higher metal and bromide content in the crude solution shows that the purification process removes excessive metal salts.

| B-site metals | Cs | Pb | Br | Sr | Ca | Cd | Mg | Br/Cs | (Pb+M)/Pb |
| --- | --- | --- | --- | --- | --- | --- | --- | --- | --- |
| PbSrCaMg | 4.7 | 9.4 | 63.3 | 15.3 | 10.1 | - | 6.1 | 13.5 | 4.4 |
| PbSrCaCd | 6.6 | 10.3 | 61.1 | 5.1 | 15.8 | 15.1 | - | 9.3 | 3.0 |
| PbCaCdMg | 4.7 | 11.1 | 64.8 | - | 16.9 | 13.6 | 6.8 | 13.8 | 3.1 |
| PbSrCaCdMg | 4.3 | 9.7 | 57.5 | 6.8 | 20.6 | 15 | 10.1 | 13.4 | 4.9 |

**Table S4.** Optical emission wavelength (λ_PL_), full width half maximum (fwhm), and carrier lifetimes (*τ*) for main parent, binary, trinary, and HEP NCs thin film studied at room temperature in this work.

| B-site metals | λ_PL_  (nm) | fwhm  (nm) | *τ*_avg_  (ns) | *τ*_1_  (ns) | *τ*_2_  (ns) | *τ*_3_  (ns) | A_1_  (-) | A_2_  (-) | A_3_  (-) |
| --- | --- | --- | --- | --- | --- | --- | --- | --- | --- |
| Pb | 516 | 20.3 | 48.46 | 6.18 | 25.30 | 120.49 | 0.62 | 0.33 | 0.04 |
| PbSr | 515 | 18.5 | 50.01 | 7.20 | 32.06 | 103.37 | 0.54 | 0.39 | 0.07 |
| PbCa | 513 | 19.4 | 36.06 | 7.82 | 27.86 | 88.07 | 0.56 | 0.39 | 0.05 |
| PbSrMg | 505 | 17.6 | 7.37 | 3.64 | 7.31 | 32.06 | 0.50 | 0.49 | 0.01 |
| PbCaMg | 509 | 18.8 | 6.68 | 3.21 | 7.21 | 34.46 | 0.80 | 0.19 | 0.01 |
| PbSrCdMg | 506 | 18.0 | 7.60 | 3.14 | 7.23 | 26.10 | 0.62 | 0.36 | 0.02 |
| PbCaCdMg | 504 | 18.4 | 6.19 | 3.52 | 8.12 | 41.49 | 0.91 | 0.08 | 0.01 |
| PbSrCaCdMg | 505 | 18.3 | 7.60 | 3.33 | 7.92 | 42.52 | 0.75 | 0.24 | 0.01 |
| Quantum-Confined CsPbBr_3_ NCs | 498 | 22 | 8.32 | 7.16 | 38.51 | - | 0.99 | 0.01 |  |

**Table S5.** TRPL Carrier lifetimes (*τ*) measured by white light LED 365 nm excitation at cryogenic conditions for the main parent, binary, trinary, and HEP NCs thin film investigated in this work. The resulting spectra are shown in **Figure S20-S23**.

**CsPbBr_3_**

| Temperature  (K) | *τ*_avg_  (ns) | *τ*_1_  (ns) | *τ*_2_  (ns) | *τ*_3_  (ns) | A_1_  (-) | A_2_  (-) | A_3_  (-) |
| --- | --- | --- | --- | --- | --- | --- | --- |
| 300 | 32.11 | 5.32 | 22.38 | 94.63 | 0.77 | 0.20 | 0.03 |
| 250 | 27.38 | 5.07 | 19.23 | 86.75 | 0.73 | 0.25 | 0.02 |
| 200 | 26.70 | 5.03 | 18.18 | 94.71 | 0.73 | 0.25 | 0.02 |
| 150 | 26.62 | 4.34 | 16.49 | 60.89 | 0.78 | 0.20 | 0.02 |
| 100 | 22.84 | 3.10 | 13.16 | 75.69 | 0.80 | 0.18 | 0.02 |
| 77 | 22.84 | 1.95 | 8.31 | 48.20 | 0.66 | 0.29 | 0.05 |

**Cs(PbSr)Br_3_**

| Temperature  (K) | *τ*_avg_  (ns) | *τ*_1_  (ns) | *τ*_2_  (ns) | *τ*_3_  (ns) | A_1_  (-) | A_2_  (-) | A_3_  (-) |
| --- | --- | --- | --- | --- | --- | --- | --- |
| 300 | 22.10 | 3.97 | 15.77 | 80.72 | 0.83 | 0.15 | 0.02 |
| 250 | 17.17 | 3.59 | 11.65 | 69.03 | 0.82 | 0.16 | 0.02 |
| 200 | 16.55 | 3.47 | 11.94 | 82.16 | 0.85 | 0.14 | 0.01 |
| 150 | 17.70 | 3.91 | 15.01 | 81.97 | 0.89 | 0.10 | 0.01 |
| 100 | 15.98 | 2.89 | 10.06 | 66.03 | 0.85 | 0.14 | 0.01 |
| 77 | 12.32 | 1.81 | 7.69 | 49.78 | 0.87 | 0.12 | 0.01 |

**Cs(PbCa)Br_3_**

| Temperature  (K) | *τ*_avg_  (ns) | *τ*_1_  (ns) | *τ*_2_  (ns) | *τ*_3_  (ns) | A_1_  (-) | A_2_  (-) | A_3_  (-) |
| --- | --- | --- | --- | --- | --- | --- | --- |
| 300 | 22.25 | 5.61 | 17.98 | 55.82 | 0.61 | 0.34 | 0.05 |
| 250 | 22.28 | 5.41 | 17.67 | 54.63 | 0.56 | 0.38 | 0.06 |
| 200 | 21.72 | 4.56 | 17.13 | 63.55 | 0.71 | 0.26 | 0.03 |
| 150 | 19.43 | 2.86 | 11.46 | 57.13 | 0.76 | 0.21 | 0.03 |
| 100 | 14.34 | 1.93 | 8.57 | 53.11 | 0.85 | 0.14 | 0.01 |
| 77 | 11.38 | 1.77 | 7.03 | 40.16 | 0.85 | 0.14 | 0.01 |

**Cs(PbSrMg)Br_3_**

| Temperature  (K) | *τ*_avg_  (ns) | *τ*_1_  (ns) | *τ*_2_  (ns) | *τ*_3_  (ns) | A_1_  (-) | A_2_  (-) | A_3_  (-) |
| --- | --- | --- | --- | --- | --- | --- | --- |
| 300 | 18.19 | 5.95 | 16.05 | 60.93 | 0.72 | 0.26 | 0.02 |
| 250 | 9.51 | 3.78 | 10.64 | 44.17 | 0.82 | 0.17 | 0.01 |
| 200 | 7.12 | 2.28 | 6.10 | 25.80 | 0.75 | 0.23 | 0.02 |
| 150 | 6.12 | 1.55 | 5.37 | 25.79 | 0.84 | 0.15 | 0.01 |
| 100 | 3.68 | 0.41 | 2.09 | 12.44 | 0.73 | 0.26 | 0.02 |
| 77 | 1.79 | 0.44 | 1.38 | 8.42 | 0.61 | 0.38 | 0.01 |

**Cs(PbCaMg)Br_3_**

| Temperature  (K) | *τ*_avg_  (ns) | *τ*_1_  (ns) | *τ*_2_  (ns) | *τ*_3_  (ns) | A_1_  (-) | A_2_  (-) | A_3_  (-) |
| --- | --- | --- | --- | --- | --- | --- | --- |
| 300 | 16.50 | 5.35 | 14.49 | 54.19 | 0.70 | 0.27 | 0.03 |
| 250 | 10.48 | 3.98 | 11.22 | 46.06 | 0.81 | 0.18 | 0.01 |
| 200 | 6.96 | 2.10 | 5.84 | 25.51 | 0.84 | 0.15 | 0.01 |
| 150 | 6.10 | 1.54 | 5.33 | 25.27 | 0.84 | 0.15 | 0.01 |
| 100 | 3.94 | 1.12 | 4.13 | 27.00 | 0.90 | 0.09 | 0.01 |
| 77 | 2.49 | 0.98 | 3.20 | 19.53 | 0.92 | 0.07 | 0.01 |

**Cs(PbSrCdMg)Br_3_**

| Temperature  (K) | *τ*_avg_  (ns) | *τ*_1_  (ns) | *τ*_2_  (ns) | *τ*_3_  (ns) | A_1_  (-) | A_2_  (-) | A_3_  (-) |
| --- | --- | --- | --- | --- | --- | --- | --- |
| 300 | 12.06 | 3.90 | 11.30 | 44.42 | 0.67 | 0.31 | 0.02 |
| 250 | 7.51 | 3.08 | 8.26 | 31.85 | 0.80 | 0.19 | 0.01 |
| 200 | 4.65 | 2.12 | 5.80 | 25.24 | 0.84 | 0.14 | 0.02 |
| 150 | 3.63 | 1.82 | 5.54 | 21.29 | 0.92 | 0.06 | 0.02 |
| 100 | 1.80 | 1.33 | 4.35 | 17.01 | 0.98 | 0.01 | 0.01 |
| 77 | 1.67 | 1.24 | 2.44 | 14.51 | 0.91 | 0.08 | 0.01 |

**Cs(PbCaCdMg)Br_3_**

| Temperature  (K) | *τ*_avg_  (ns) | *τ*_1_  (ns) | *τ*_2_  (ns) | *τ*_3_  (ns) | A_1_  (-) | A_2_  (-) | A_3_  (-) |
| --- | --- | --- | --- | --- | --- | --- | --- |
| 300 | 9.24 | 3.42 | 8.47 | 53.96 | 0.83 | 0.16 | 0.1 |
| 250 | 4.46 | 2.18 | 4.34 | 21.04 | 0.74 | 0.25 | 0.01 |
| 200 | 2.32 | 1.66 | 7.68 | - | 0.97 | 0.03 | - |
| 150 | 1.63 | 1.23 | 5.88 | - | 0.98 | 0.02 | - |
| 100 | 1.24 | 1.05 | 4.53 | - | 0.99 | 0.01 | - |
| 77 | 1.39 | 1.07 | 2.47 | - | 0.89 | 0.11 | - |

**Cs(PbSrCaCdMg)Br_3_**

| Temperature  (K) | *τ*_avg_  (ns) | *τ*_1_  (ns) | *τ*_2_  (ns) | *τ*_3_  (ns) | A_1_  (-) | A_2_  (-) | A_3_  (-) |
| --- | --- | --- | --- | --- | --- | --- | --- |
| 300 | 17.58 | 4.28 | 15.69 | 76.45 | 0.86 | 0.13 | 0.01 |
| 250 | 10.30 | 3.08 | 10.71 | 56.00 | 0.91 | 0.08 | 0.01 |
| 200 | 2.84 | 1.78 | 5.57 | 32.93 | 0.96 | 0.03 | 0.01 |
| 150 | 1.99 | 1.56 | 9.50 | - | 0.99 | 0.01 | - |
| 100 | 1.36 | 1.10 | 5.56 | - | 0.98 | 0.02 | - |
| 77 | 1.18 | 1.02 | 2.05 | - | 0.92 | 0.08 | - |

**Table S6**. Global fitting parameters of fluence-dependent TA GSB kinetics of samples with different alloyed elements.

| Samples | τ_1_ (ns) | τ_2_ (ps) | τ_3_ (ps) |
| --- | --- | --- | --- |
| Parent | 5.4 ± 0.3 | 95 ± 4 | 510 ± 40 |
| PbSr | 5.8 ± 0.2 | 77 ± 3 | 410 ± 20 |
| PbSrCa | 4.3 ± 0.1 | 53 ± 3 | 240 ± 30 |
| PbSrMg | 3.8 ± 0.1 | 45 ± 4 | 170 ± 50 |
| PbSrCd | 3.6 ± 0.1 | 34 ± 2 | 230 ± 90 |
| PbSrCaMg | 3.7 ± 0.1 | 47 ± 5 | 160 ± 50 |
| PbSrCdMg | 4.1 ± 0.1 | 37 ± 2 | 320 ± 80 |
| PbSrCaCdMg | 4.5 ± 0.1 | 46 ± 3 | 210 ± 30 |

**References**

(1) Solari, S. F.; Poon, L.-N.; Wörle, M.; Krumeich, F.; Li, Y.-T.; Chiu, Y.-C.; Shih, C.-J. Stabilization of lead-reduced metal halide perovskite nanocrystals by high-entropy alloying. *Journal of the American Chemical Society* **2022**, *144* (13), 5864-5870.

(2) Boehme, S. C.; Bodnarchuk, M. I.; Burian, M.; Bertolotti, F.; Cherniukh, I.; Bernasconi, C.; Zhu, C.; Erni, R.; Amenitsch, H.; Naumenko, D.; Andrusiv, H.; Semkiv, N.; Abraham John, R.; Baldwin, A.; Galkowski, K.; Masciocchi, N.; D. Stranks, S.; Rainò, G.; Guagliardi, A.; Kovalenko, M. V. Strongly confined CsPbBr3 quantum dots as quantum emitters and building blocks for rhombic superlattices. *ACS nano* **2023**, *17* (3), 2089-2100.

(3) Wieczorek, A.; Kuba, A. G.; Sommerhäuser, J.; Caceres, L. N.; Wolff, C. M.; Siol, S. Advancing high-throughput combinatorial aging studies of hybrid perovskite thin films via precise automated characterization methods and machine learning assisted analysis. *Journal of Materials Chemistry A* **2024**, *12* (12), 7025-7035.

(4) Kresse, G.; Furthmüller, J. Efficient iterative schemes for ab initio total-energy calculations using a plane-wave basis set. *Physical review B* **1996**, *54* (16), 11169.

(5) Perdew, J. P.; Burke, K.; Ernzerhof, M. Generalized gradient approximation made simple. *Physical review letters* **1996**, *77* (18), 3865.

(6) Monkhorst, H. J.; Pack, J. D. Special points for Brillouin-zone integrations. *Physical review B* **1976**, *13* (12), 5188.

(7) Leong, Z.; Tan, T. L. Robust cluster expansion of multicomponent systems using structured sparsity. *Physical Review B* **2019**, *100* (13), 134108.

(8) Feldmann, S.; Gangishetty, M. K.; Bravić, I.; Neumann, T.; Peng, B.; Winkler, T.; Friend, R. H.; Monserrat, B.; Congreve, D. N.; Deschler, F. Charge carrier localization in doped perovskite nanocrystals enhances radiative recombination. *Journal of the American Chemical Society* **2021**, *143* (23), 8647-8653.

(9) Ahmed, G. H.; Liu, Y.; Bravić, I.; Ng, X.; Heckelmann, I.; Narayanan, P.; Fernández, M. S.; Monserrat, B.; Congreve, D. N.; Feldmann, S. Luminescence enhancement due to symmetry breaking in doped halide perovskite nanocrystals. *Journal of the American Chemical Society* **2022**, *144* (34), 15862-15870.
